# An Auto‐Activated NLR‐Protein OsRGA3^D605V^ Confers Rice Triple Resistance and Deactivates Resistance After Phosphorylation by OsILA1

**DOI:** 10.1111/pbi.70471

**Published:** 2025-11-27

**Authors:** Yuan Zhong, Su Chen, Bo Sun, Min Liu, Zhenying Shi, Xuexia Miao, Haichao Li

**Affiliations:** ^1^ Key Laboratory of Plant Design, CAS Center for Excellence in Molecular Plant Sciences, Institute of Plant Physiology and Ecology Chinese Academy of Sciences Shanghai China; ^2^ University of Chinese Academy of Sciences Beijing China; ^3^ Chongming Agricultural Technology Extension Center Shanghai China

**Keywords:** bacterial leaf blight, brown planthopper, hypersensitive response, MHD motif, OsILA1, OsRGA3, Raf‐like MAPKKK, rice blast disease, rice immunity

## Abstract

The incidence of pests and diseases seriously impacts rice production, and *NLR* genes play a crucial role in the regulation of immune signalling in rice. Here, we identified an *NLR* gene *OsRGA3* that positively regulates rice resistance to brown planthopper (BPH) and rice blast disease (RBD). The mutant OsRGA3^D605V^ as an auto‐activated form of OsRGA3 can form a resistosome and exhibit Ca^2+^ permeable channel activity, triggering a hypersensitive response (HR) and providing rice with enhanced resistance to BPH, RBD and bacterial leaf blight (BLB). Biochemistry experiments confirmed that a Raf‐like MAPKKK OsILA1 interacts with OsRGA3 by Y2H, LCA, Pull‐down and BLI assay. OsILA1 negatively regulates rice resistance to BPH, RBD and BLB. Further study discovered that OsILA1 inhibits HR triggered by OsRGA3^D605V^ through phosphorylation of Y15 and Y138 sites to avoid an excessive immune response of plants. This study discovered a new MAPK‐NLR module with triple bio‐stress resistance that can be self‐activated and then deactivated by phosphorylation.

## 1 Introduction

Over the years, the prevalence and devastation of pests and diseases have posed a significant challenge to rice cultivation. Among these, the brown planthopper (BPH), rice blast disease (RBD) and bacterial leaf blight (BLB) stand out as particularly destructive, inflicting substantial annual yield losses across rice‐growing regions (Ji et al. 2016; Mew 1987; Qiu et al. 2012; Saleh et al. 2014; Xiao et al. 2016). BPH utilises its piercing‐sucking mouthparts to extract sap from the phloem of rice plants, while also transmitting rice diseases. RBD adheres to rice leaves via spores, then spreads intercellularly to absorb nutrients, resulting in varying degrees of disease spots and necrosis on the leaves, stems, and panicles of rice plants. BLB is characterised by its rapid spread and primarily targets rice leaves, causing extensive white necrosis. Leaves, being the principal organs for photosynthesis, suffer damage from pathogens that impair the plants' photosynthetic processes, leading to a decrease in the production of organic matter and consequently affecting the growth and yield of rice. The development of resistant varieties represents the most cost‐effective, efficient, and environmentally friendly approach to pest and disease control. To date, 45 BPH resistance genes, over 100 RBD resistance genes, and at least 47 BLB resistance genes have been identified, laying a theoretical foundation for resistance breeding (Simon et al. 2023). Nevertheless, the diversity and rapid mutation of BPH, RBD, and BLB mean that the resistance of these varieties often wanes or is lost after a few years of deployment. Thus, the ongoing quest to uncover rice resistance genes is vital, as it offers theoretical guidance and germplasm resources for rice resistance breeding, playing a critical role in ensuring the safety and sustainability of rice production.

In response to the invasion of diverse pathogens, plants have developed an elaborate immune machinery. Including the nucleotide‐binding site (NBS) and leucine‐rich repeat (LRR) domains, NLRs are integral components of the plant immune system (Belkhadir et al. 2004; Kourelis and van der Hoorn 2018; Wilmanski et al. 2008), which can directly or indirectly perceive the pathogen effectors and play a pivotal role in the transduction of immune signals (Dangl and Jones 2001; Dodds and Rathjen 2010). It has been reported that the rice NLR protein BPH14 can recognise BPH salivary protein BISP through direct binding, thereby activating the rice immune response (Guo et al. 2023). The NLR protein RGA5 recognises rice blast effectors AVR‐Pia and AVR1‐CO39 through direct binding and, in conjunction with the NLR RGA4, confers resistance to RBD in rice (Cesari et al. 2013). However, NLRs that function simultaneously in the plant's defense against both BPH and RBD have not yet been reported.

The N‐terminal conserved structural domains of NLR proteins are three types: the Toll/interleukin‐1 receptor (TIR) domain, the coiled‐coil (CC) domain, and the Resistance to Powdery Mildew 8 (RPW8) domain. The CC and TIR domains play crucial roles in protein–protein interactions and are key to NLR oligomerization (Bi et al. 2021). The LRR domain has lower conservation and is an important determinant of protein specificity (Ellis and Jones 1998), inhibiting NLR activity in the resting state (Ade et al. 2007). The NBS domain interacts with ADP or ATP, regulating the resting or activated state of NLRs (Steele et al. 2019). The NBS contains three subdomains: NB‐ARC, ARC1, and ARC2, with numerous highly conserved motifs (Lukasik and Takken 2009; Takken et al. 2006). The MHD motif within ARC2 acts as a phosphate sensor, participating in nucleotide‐dependent conformational changes (Van Ooijen et al. 2008), and mutations in the MHD motif can lead to auto‐activation of various NLR proteins (Bendahmane 2003; Gao et al. 2011; Howles et al. 2005; Van Ooijen et al. 2008). Upon effectors recognition or auto‐activation, NLRs form inflammasomes or acquire enzymatic activity, thereby inducing a hypersensitive response (HR) in tissues. Studies have shown that upon activation, the NLR ZAR1 changes the conformation of the CC domain, oligomerizes into a pentameric complex acting as Ca^2+^ permeable channels to trigger calcium influx, which leads to HR (Bi et al. 2021). NLRs play a crucial role in plant immunity, and the study of NLRs is indispensable for elucidating resistance mechanisms in rice and the development of resistant varieties.

In plants, Mitogen‐activated protein kinase (MAPK) cascades are highly conserved signalling modules that comprise MAPK Kinase Kinases (MAPKKKs/MEKKs), MAPK Kinases (MAPKKs/MKKs/MEKs), and MAPKs/MPKs. These kinases transmit signals through phosphorylation and play a significant role in plant immunity (Devendrakumar et al. 2018; Sun and Zhang 2022). For instance, in *Arabidopsis*, WRKY33 can be phosphorylated by MPK4 and MPK6, thereby responding to *Botrytis cinerea* infection (Mao et al. 2011). Studies have indicated that plant MAPK pathways are associated with the HR, such as the silencing of MAPKKKα or MAPKKKε in tobacco, which inhibits the HR triggered by pathogen‐activated resistance proteins, thereby regulating plant immunity (del Pozo et al. 2004; Melech‐Bonfil and Sessa 2010). Silencing of the soybean *MAPK KINASE KINASE1 (GmMEKK1)* results in HR and enhances resistance to downy mildew (*Peronospora manshurica*) and *Soybean mosaic* virus (Xu et al. 2018). Silencing of *NbMAP3Kβ2* in tobacco enhances the colonisation of *P. infestans* and attenuates Cf4/Avr4‐induced cell death (Ren et al. 2019). *OsACDRT* encodes a putative Raf‐like MAPKKK, and overexpression of *OsACDRT* in rice leads to spontaneous HR‐like lesions in leaves and confers resistance to RBD (Kim et al. 2009). Whether there is a direct relationship between the MAPK pathway and NLRs in rice has not yet been reported.

In this study, we discovered that OsRGA3 confers rice resistance to BPH and RBD. OsRGA3^D605V^ as an auto‐activated form of OsRGA3, confers stronger and more broad‐spectrum rice resistance to BPH, RBD and BLB, but affects rice growth and yield. Further study confirmed that a Raf‐like MAPKKK OsILA1 can directly interact with OsRGA3, phosphorylating it to impede its plasma membrane (PM) localisation, thereby inhibiting the function of OsRGA3^D605V^ and negatively regulating rice resistance to BPH, RBD and BLB.

## 2 Result

### 2.1 An NLR Protein OsRGA3 Positively Regulates Rice Resistance to BPH and RBD, but Not to BLB

**FIGURE 1:**
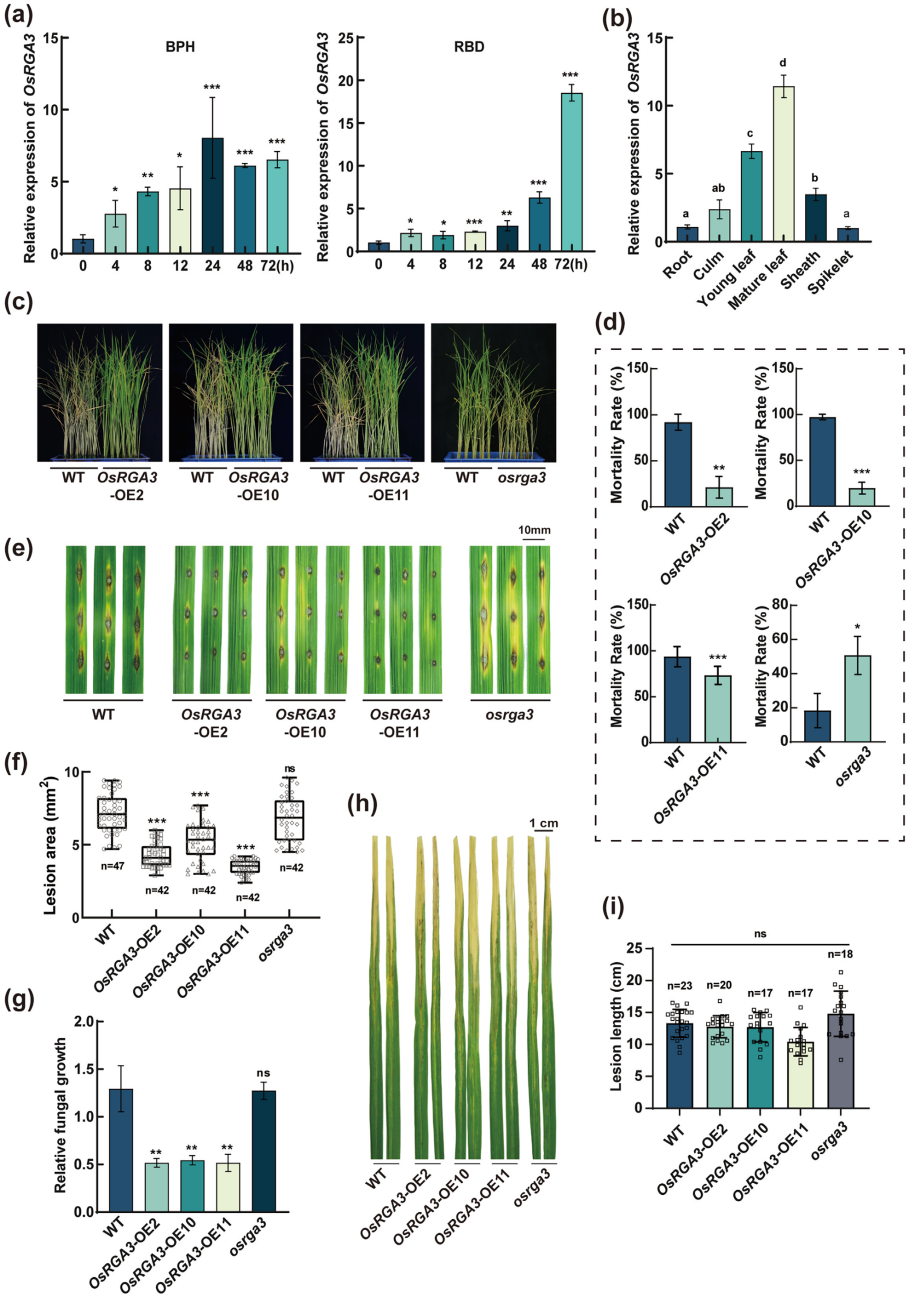
*OsRGA3* positively regulates rice resistance to BPH and RBD. (a, b) Result of the qRT‐PCR analysis of *OsRGA3* transcription in rice during BPH infestation and RBD infection (a), and in different rice tissues (b). (c, d) The plant status (c) and survival rate (d) of OsRGA3 transgenic lines verse WT plants after BPH infestation in small population test. (e–g) Phenotypes (e), lesion area (f) and relative fungal growth (g) of OsRGA3 transgenic lines at 7 days after a punch inoculation with isolate TH12. The copy number of *MoPot2* in TH12 on the punch‐inoculation leaves was measured to quantify relative fungal growth in plants. (h, i) Phenotypes (h) and lesion area (i) of OsRGA3 transgenic lines at 7 days after inoculated with BLB. Data in (f) are displayed as box and whisker plots with individual data points. The error bars represent maximum and minimum values. Center line, median; box limits, 25th and 75th percentiles. ***p < 0.001 (two‐tailed *t*‐test, compared to WT). Error bars in (a), (b), (d), (g) and (i) indicate ± SD (*n* = 3). *p < 0.05, **p < 0.01, ***p < 0.001 (two‐tailed *t*‐test, compared to 0 h or WT).

We pinpointed *OsRGA3*, an *NLR* gene linked to rice resistance, by intersecting mutant‐library inserts with prior Pia mapping and transcriptome data (Okuyama et al. 2011; Tian et al. 2020; Zhang et al. 2015). Through a BLAST analysis of the OsRGA3 amino acid sequence, we identified its homologous protein, RPM1, in *Arabidopsis*. The results indicated that OsRGA3 encodes a CC‐NBS‐LRR protein, which shares the same key motifs as RPM1, including EDVIV, P‐LOOP, NBS‐A, Walker‐B, NBS‐C, GLPL, RNBS‐D, and MHD (Figure S1). Moreover, we analyzed the evolutionary relationships between OsRGA3 and other rice NLR proteins and constructed a phylogenetic tree. The results showed that OsRGA3 had a closer evolutionary relationship with Pid3, which confers RBD resistance (Figure S2) (Zhao et al. 2016; Zhou et al. 2019). Under BPH and RBD infection, the expression of *OsRGA3* was up‐regulated significantly (Figure 1a). The expression levels in sheath and leaves of rice were significantly higher than those in root and spikelet (Figure 1b). The rice sheath and leaves were the main tissues damaged by BPH and RBD, suggesting that *OsRGA3* may be involved in rice immune regulation in response to these biotic stresses.

To further investigate the function of *OsRGA3*, we constructed the *OsRGA3* knockout line *osrga3*, and overexpression lines *OsRGA3*‐OE2, *OsRGA3*‐OE10, and *OsRGA3*‐OE11, respectively (Figure S3a,b). The growth and development phenotypes of all transgenic strains are shown in Figure S3c,d. *OsRGA3* did not alter tillering‐stage height or 1000‐grain weight, whereas *osrga3* exhibited a significant decrease in the panicle seed setting rate. Through RT‐qPCR detection, we found that the expression of immunoregulation‐related genes *OsPR5*, *OsPR10a* and *OsJAZ8* was significantly up‐regulated in *OsRGA3*‐OE lines compared with WT (Figure S3e). The three *OsRGA3*‐OE lines exhibited significant resistance to BPH and RBD, while *osrga3* showed a susceptible phenotype to BPH (Figure 1c–g; Figure S3f). In addition, *OsRGA3* is irrelevant in the process of BLB harming rice (Figure 1h,i). These results indicated that *OsRGA3* positively regulates rice resistance to BPH and RBD, but not to BLB.

### 2.2 OsRGA3^D605V^ Can Automatically Activate Hypersensitive Response and Enhances Rice Resistance to BPH, RBD and BLB

**FIGURE 2:**
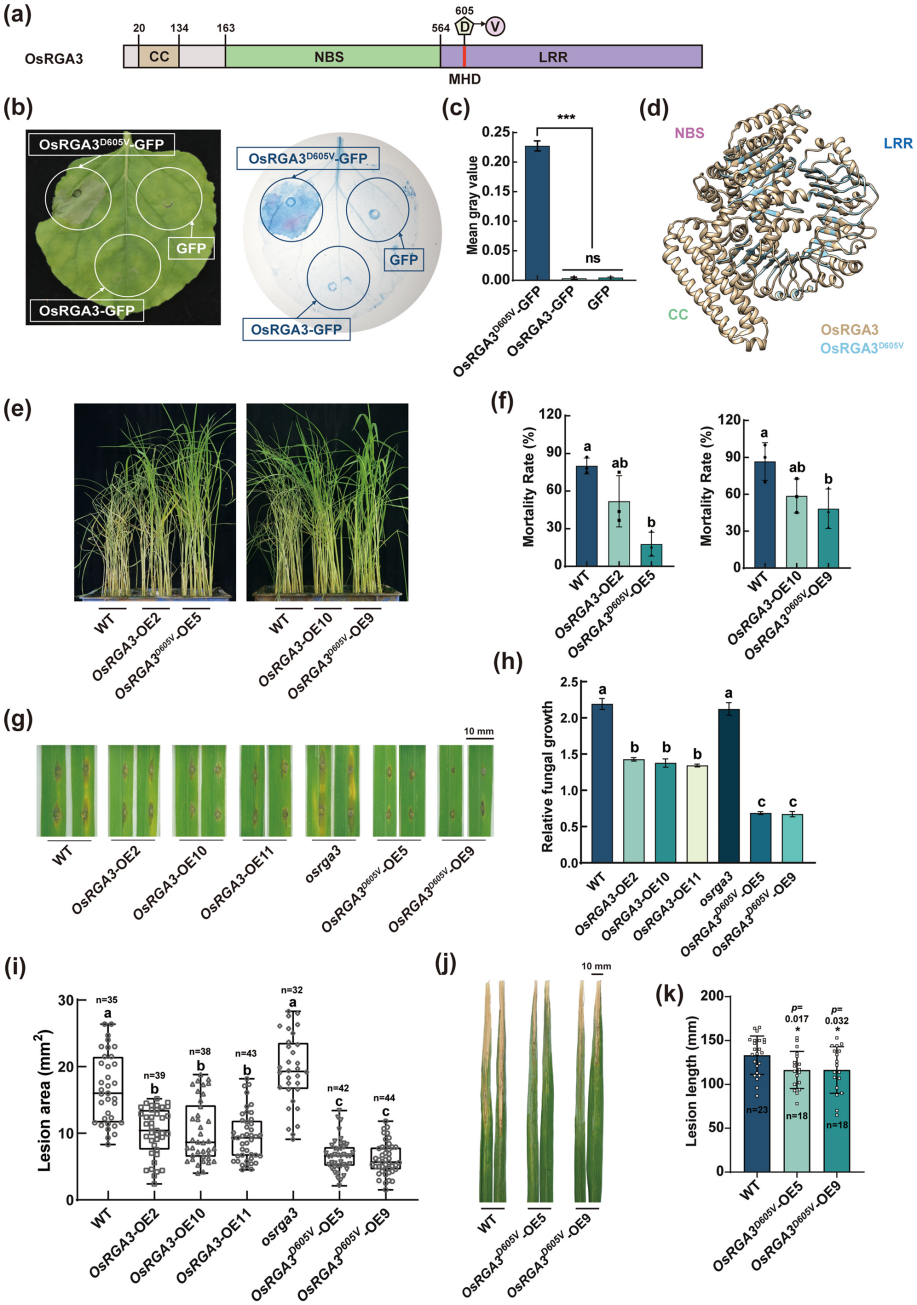
OsRGA3^D605V^ can autoactivate HR and confers enhanced rice resistance to BPH, RBD and BLB by MHD motif mutations. (a) Schematic diagram of OsRGA3 domains and MHD motif. (b) Transient expression of OsRGA3^D605V^‐GFP, OsRGA3‐GFP, and GFP in *Nb* leaves and stained with trypan blue. (c) The grey value statistical results involved in (b). (d) The predicted three‐dimensional structural superimposition results of the OsRGA3 and OsRGA3^D605V^ proteins, with yellow protein representing OsRGA3 and blue protein representing OsRGA3^D605V^. (e, f) The plant status (e) and the survival rate (f) of WT, OsRGA3‐OE2, OE10, and OsRGA3^D605V^‐OE5, OE9 plants after BPH infestation in small population test. (g–i) Phenotypes (g), lesion area (h) and relative fungal growth (i) of OsRGA3^D605V^ transgenic lines plants at 7 days after a punch inoculation with isolate TH12. (j, k) Phenotypes (j) and lesion area (k) of OsRGA3^D605V^ transgenic lines plants at 7 days after inoculated with BLB. Data in (h) are displayed as box and whisker plots with individual data points. The error bars represent maximum and minimum values. Center line, median; box limits, 25th and 75th percentiles. The *p* values were determined by one‐way ANOVA with two‐sided Tukey's HSD test and indicated with different letters, *p* < 0.01. Error bars in (c), (f), (i) and (k) indicate ± SD (*n* = 3). The *p* values in (c) and (k) were determined by two‐tailed unpaired Student's *t*‐test, *p < 0.05, ***p < 0.001. The *p* values in (f) and (i) were determined by one‐way ANOVA with two‐sided Tukey's HSD test and indicated with different letters, *p* < 0.01.

Previous studies have shown that the mutation of the MHD motif to MHV can automatically activate RPM1, and the HR can be triggered without effector induction (Gao et al. 2011). For OsRGA3, we mutated the MHD motif to MHV (Figure 2a), and then the 35S‐*OsRGA3* ^*D605V*^‐GFP vector was constructed and transformed into tobacco (*Nicotiana benthamiana*, *Nb*), showing that OsRGA3^D605V^ could cause HR (Figure 2b). Consistently, the grey value of leaves expressing *OsRGA3* ^*D605V*^ was significantly higher than that of the control group by Trypan blue staining (Figure 2c). Through predicting the three‐dimensional structure of the OsRGA3 and OsRGA3^D605V^ proteins, we found that the NBS and LRR domains of OsRGA3^D605V^ were significantly changed compared with OsRGA3 (Figure 2d), suggesting that the structural changes in OsRGA3 occur in response to an attack by invaders, leading to the formation of an active state.

To further investigate the impact of the MHD mutation on the function of the OsRGA3 protein, we constructed overexpression lines *OsRGA3* ^*D605V*^‐OE5 and *OsRGA3* ^*D605V*^‐OE9. The expression of OsRGA3 and immunoregulation‐related genes *OsJAZ8*, OsJAZ11, *OsPR5* is significantly upregulated in *OsRGA3* ^*D605V*^‐OE lines (Figure S4a). Field‐based statistical analysis showed that *OsRGA3* ^*D605V*^ overexpression significantly reduced plant height at tillering and the panicle seed‐setting rate but had no effect on thousand‐grain weight (Figure S4b,c). *OsRGA3* ^*D605V*^‐OE lines exhibited resistance phenotypes against BPH (Figure S5) and showed a stronger resistance phenotype than *OsRGA3*‐OE lines (Figure 2e,f). Similarly, the *OsRGA3* ^*D605V*^‐OE lines exhibited a more pronounced resistance phenotype against RBD than *OsRGA3*‐OE lines (Figure 2g–i). Furthermore, *OsRGA3* ^*D605V*^‐OE lines display stronger resistance to BLB contrasted with the wild type, which is a characteristic phenotype not observed in *OsRGA3*‐OE lines (Figure 2j,k). The above results demonstrate that compared with *OsRGA3*‐OE lines, *OsRGA3* ^*D605V*^‐OE lines endow rice with augmented broad‐spectrum resistance to biotic stress.

### 2.3 OsRGA3 and OsRGA3^D605V^ Exhibit Ca^2+^ Permeable Channel Activity to Trigger Plant Immune Responses

**FIGURE 3:**
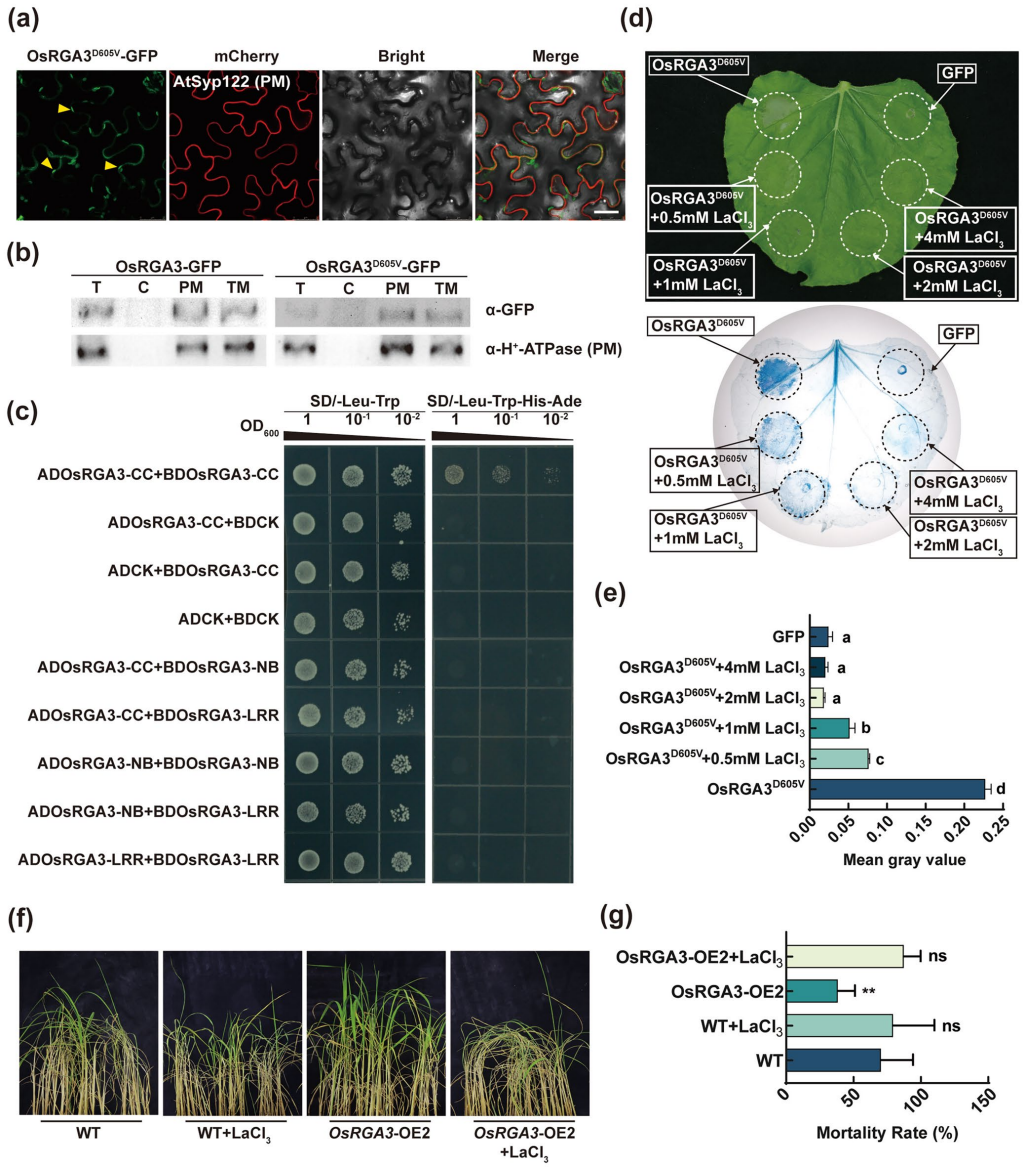
LaCl_3_ inhibits the immune response triggered by OsRGA3^D605V^. (a) Confocal microscopy analyses of OsRGA3^D605V^. Bars, 25 μm. (b) Membrane fractionation assays were used to assess the PM localisation of OsRGA3 and OsRGA3^D605V^. (c) The Y2H assay examines the interaction between the OsRGA3 domains. (d) OsRGA3^D605V^‐GFP with the increase of LaCl_3_ concentration is expressed transiently in *Nb* leaves and stained with trypan blue. (e) The grey value statistical results after trypan blue staining of *Nb* leaves in (d). (f, g) The plant status (f) and survival rate (g) of OsRGA3 transgenic lines verse WT plants after BPH infestation and treatment with LaCl_3_ in small population test. Error bars in (e) and (g) indicate ± SD (*n* = 3). The *p* values in (e) were determined by one‐way ANOVA with two‐sided Tukey's HSD test and indicated with different letters, *p* < 0.01. The *p* values in (g) were determined by two‐tailed unpaired Student's *t*‐test, **p < 0.01.

It has been reported that the ZAR1 resistosome acts as Ca^2+^ permeable channels to trigger immunity and cell death (Bi et al. 2021). We found that OsRGA3^D605V^ can form puncta on the PM through subcellular localisation experiments (Figure 3a). We performed plasma‐membrane (PM) enrichment by differential and density‐gradient centrifugation. Western blotting with anti‐GFP and anti‐PM H^+^‐ATPase antibodies confirmed that both OsRGA3 and OsRGA3^D605V^ reside almost exclusively at the PM, with negligible signal in the cytosol (Figure 3b). The Y2H experiments showed that the CC domain of OsRGA3 could interact with itself, but there was no interaction between other domains (Figure 3c) indicating OsRGA3 can form a typical resistosome. In addition, HR caused by OsRGA3^D605V^ was inhibited with the increased concentration of calcium channel blocker LaCl_3_ in *Nb* and almost disappeared when the concentration reached 2 mM (Figure 3d,e). Meanwhile, the BPH resistant phenotype disappeared in *OsRGA3*‐OE lines sprayed with 2 mM LaCl_3_ solution, and the rice mortality was consistent with that of the wild type (Figure 3f,g), suggesting that OsRGA3 induces immune response through the similar mechanism between HR in tobacco and the resistant phenotype in rice. Above all, OsRGA3 and OsRGA3^D605V^ exhibit Ca^2+^ permeable channel activity and may function as Ca^2+^ permeable channels through oligomerization to form polymers, which mediate immune responses.

### 2.4 Autoactivated OsRGA3^D605V^ Mediated Immune Response Requires the Full‐Length of Protein

**FIGURE 4:**
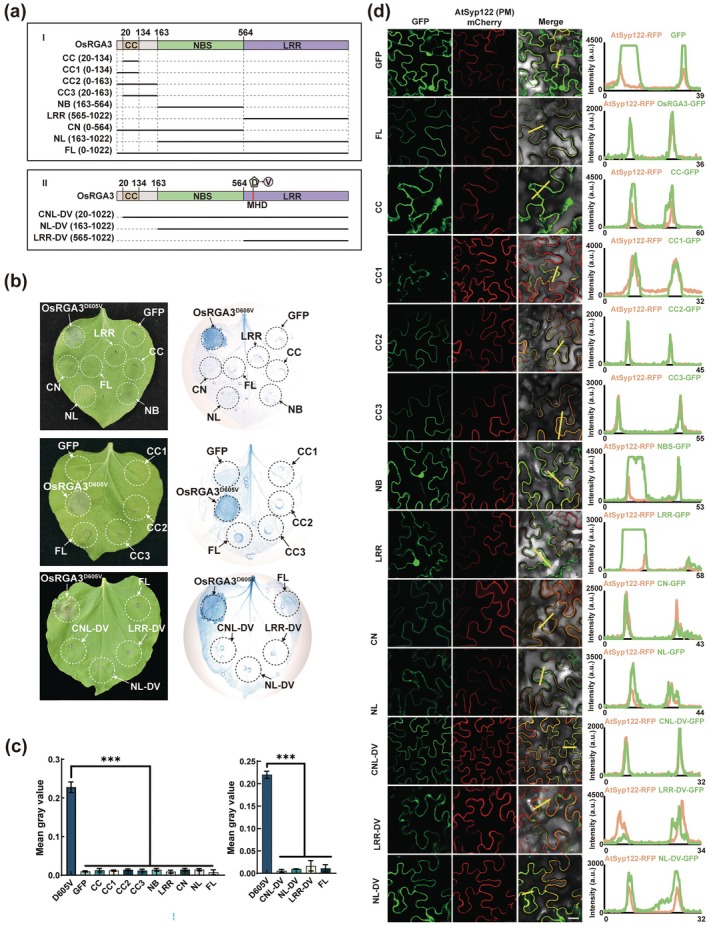
The function of OsRGA3 requires full‐length protein. (a) The OsRGA3 and OsRGA3^D605V^ proteins were divided into different fragments. (b) The different fragments from OsRGA3 and OsRGA3^D605V^ expressed transiently in *Nb* leaves and stained with trypan blue. (c) The grey value statistical results after trypan blue staining of *Nb* leaves expressing the different fragments from OsRGA3 and OsRGA3^D605V^. (d) Confocal microscopy analyses of the different fragments from OsRGA3 and OsRGA3^D605V^. Error bars in (c) indicate ± SD (*n* = 3). The *p* values were determined by two‐tailed unpaired Student's *t*‐test, ***p < 0.001. The indicated proteins in (d) fused with a C‐terminal GFP were transiently coexpressed with the PM marker AtSyp122 fused to mCherry in *Nb* leaves, and confocal images were taken at 48 to 72 h post infiltration. Confocal images are single plane secant views. Merge means merged image between GFP and mCherry images. Fluorescence intensities were measured along the yellow line depicted in the merge images. Bars, 25 μm.

Considering that OsRGA3 showed protein domain polymorphisms in different rice varieties, we further investigate the function of each domain for this NLR protein. Then, OsRGA3 and OsRGA3^D605V^ proteins were divided into different fragments and expressed in tobacco *Nb* (Figure 4a,b). All fragments except full‐length OsRGA3^D605V^ did not cause HR, and the first 20 amino acids in the N‐terminal before the CC domain were necessary for OsRGA3^D605V^ to induce HR. After Trypan blue staining, grey statistical results showed that there was no significant difference between each fragment and the negative control group (Figure 4c). Furthermore, 72 h later, there was no significant difference in calcium ion concentration in tissues expressing different fragments when compared to the negative control, except for OsRGA3^D605V^ (Figure S6).

Through online analysis of *OsRGA3* gene sequences within the genomes of different rice varieties (snp‐seek.irri.org), it was found that, like *BPH1/9*, *OsRGA3* was less conserved at multiple sites (Figure S7a). The alignment of amino acid sequences indicated that, compared with the japonica rice varieties of ZH11 and NIP, three BPH susceptibility indica rice varieties of 9311, MH63 and ZS97 have missed the N‐terminal CC domain including α1 and α2 helix, and a BPH resistant indica rice variety RHT has missed the C‐terminal LRR domain (Figure S7b). The three‐dimensional structure for OsRGA3 protein showed that LRR and NBS domain form a pocket, that can bind to ATP (Bonardi et al. 2012), but the OsRGA3 from RHT is unable to form a pocket due to the absence of the LRR domain (Figure S7c). Compared with ZH11 and NIP, RHT carries a single‐base deletion (T) at position 6 533 911 of OsRGA3, generating a premature stop codon and a truncated protein. To survey the distribution of this allele, we re‐sequenced and aligned OsRGA3 in 3000 diverse rice accessions. As illustrated in Figure S8, the identical frameshift mutation is highly enriched in indica subgroups (ind1A, ind2, ind3 and indx), whereas japonica varieties rarely carry the deletion. Consistent with earlier reports that indica germplasm generally displays higher BPH resistance than japonica (Can‐xing et al. 2008), the results indicate that the loss‐of‐function allele of OsRGA3 is associated with enhanced resistance, further supporting a functional role for OsRGA3 in rice‐BPH interactions.

PM localisation is vital for OsRGA3 to function as Ca^2+^ permeable channels. Previous studies have shown that the role of NLR protein inducing HR depends on PM localisation (El Kasmi et al. 2017; Wang et al. 2023). To verify which fragment determines PM localisation of OsRGA3 and OsRGA3^D605V^, we tested the subcellular localisations of different fragments (Figure 4d). The results show that OsRGA3^FL^, OsRGA3^CC2^, OsRGA3^CC3^, OsRGA3^CN^, OsRGA3^CNL‐DV^, containing the CC domain, can localise to the PM. Conversely, the fragments without the CC domain, including OsRGA3^NB^, OsRGA3^LRR^, OsRGA3^NL^, OsRGA3^LRR‐DV^ and OsRGA3^NL‐DV^ exhibited reduced PM localisation. Remarkably, OsRGA3^CC^ and OsRGA3^CC1^, which contain the CC domain but lack amino acids at positions 135–162, also exhibited reduced PM localisation. The above results indicate that the CC domain and the 135–162 amino acids in the CC domain are crucial for the PM localisation of OsRGA3.

### 2.5 OsILA1 Interacts With OsRGA3 and Negatively Regulates Rice Resistance to Three Kinds of Biotic Stress

**FIGURE 5:**
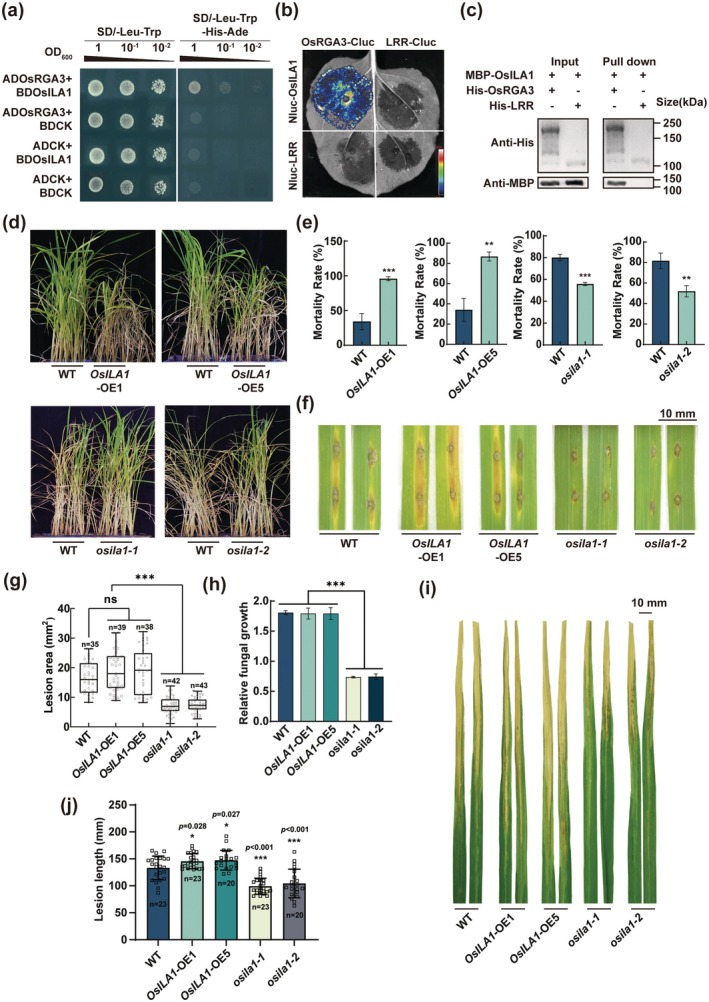
OsILA1 interacts with OsRGA3 and negatively regulates rice resistance to BPH, RBD and BLB. (a) Y2H assay demonstrates the interaction between OsRGA3 and OsILA1. (b) Luciferase complementation assay demonstrates the interaction between OsRGA3 and OsILA1 in *Nb*. The fluorescent signal intensity is indicated. (c) Pull down assay demonstrates the interaction between OsRGA3 and OsILA1 in vitro. (d, e) The plant status (d) and survival rate (e) of OsILA1 transgenic lines verse WT plants after BPH infestation in small population test. (f–h) Phenotypes (f), lesion area (g) and relative fungal growth (h) of OsILA1 transgenic lines at 7 days after a punch inoculation with isolate TH12. (i, j) Phenotypes (i) and lesion area (j) of OsILA1 transgenic lines plants at 7 days after inoculated with BLB. Data in (g) are displayed as box and whisker plots with individual data points. The error bars represent maximum and minimum values. Center line, median; box limits, 25th and 75th percentiles. The *p* values were determined by one‐way ANOVA with two‐sided Tukey's HSD test and indicated with different letters, *p* < 0.01. Error bars in (e), (h) and (i) indicate ± SD (*n* = 3). The *p* values in (e), (h) and (i) were determined by two‐tailed unpaired Student's *t*‐test, *p < 0.05, **p < 0.01, ***p < 0.001.

Through online prediction of protein interaction network (cn.string‐db.org/), we found that there may be protein interaction between rice protein OsILA1 and OsRGA3. OsILA1 is a Raf‐like MAPKKK, which can phosphorylate downstream protein MAPKK4 to regulate rice resistance to BLB (Chen, Wang, et al. 2021; Chen, Liu, et al. 2021). The expression of *OsILA1* was significantly upregulated after rice was infested by BPH, RBD and BLB (Figure S9a–c). Among different tissues of rice, the highest expression of *OsILA1* was in mature leaves (Figure S9d). OsILA1 was found to be dispersed in the cell membrane and nucleus by fluorescence microscopy (Figure S10). Subsequently, we demonstrated that OsILA1 can interact with OsRGA3 in vivo and in vitro through Y2H, LCA, Pull‐down and BLI experiments (Figure 5a–c and Figure S11). Additionally, OsILA1 could only interact with the NB domain of OsRGA3, but not with the CC and LRR domains through Y2H (Figure S12).

To further investigate the function of OsILA1, we constructed *OsILA1* knockout lines, *osila1‐1* and *osila1‐2*, and overexpression lines, OsILA1‐OE1 and OsILA1‐OE5 (Figure S13). The expression level of *OsILA1* was significantly down‐regulated, while an immune regulation‐related gene *OsAOC* was significantly up‐regulated in *osila1* lines. The opposite pattern was observed in OsILA1‐OE lines (Figure S14b). Field‐based statistical analysis showed that the overexpression of *OsILA1* significantly increased tillering‐stage height and decreased 1000‐grain weight, with no effect on the panicle seed setting rate; knockout lines were indistinguishable from wild type (Figure S15).

Further test results revealed that *osila1* lines showed a resistance phenotype to BPH, RBD and BLB, with significantly lower BPH mortality rates, RBD lesion areas and a shorter length of BLB than the wild type. In contrast, *OsILA1*‐OE lines showed an opposite phenotype (Figure 5d–j), indicating that OsILA1 negatively regulates rice resistance to these three biotic stresses. We quantified calcium ion concentrations in the leaf sheath tissues of WT and resistant lines before and after BPH infestation (Figure S15a). Before BPH infestation, *OsRGA3*‐OE9 showed markedly higher cytosolic Ca^2+^ levels than all other lines; after BPH infestation, resistant lines displayed a significant Ca^2+^ surge, which supplied additional support for the conclusion that OsRGA3 functions as Ca^2+^ permeable channels.

### 2.6 OsILA1 Inhibits Immune Responses Triggered by OsRGA3^D605V^ Through Phosphorylates at Its Y15 and Y138 Sites

**FIGURE 6:**
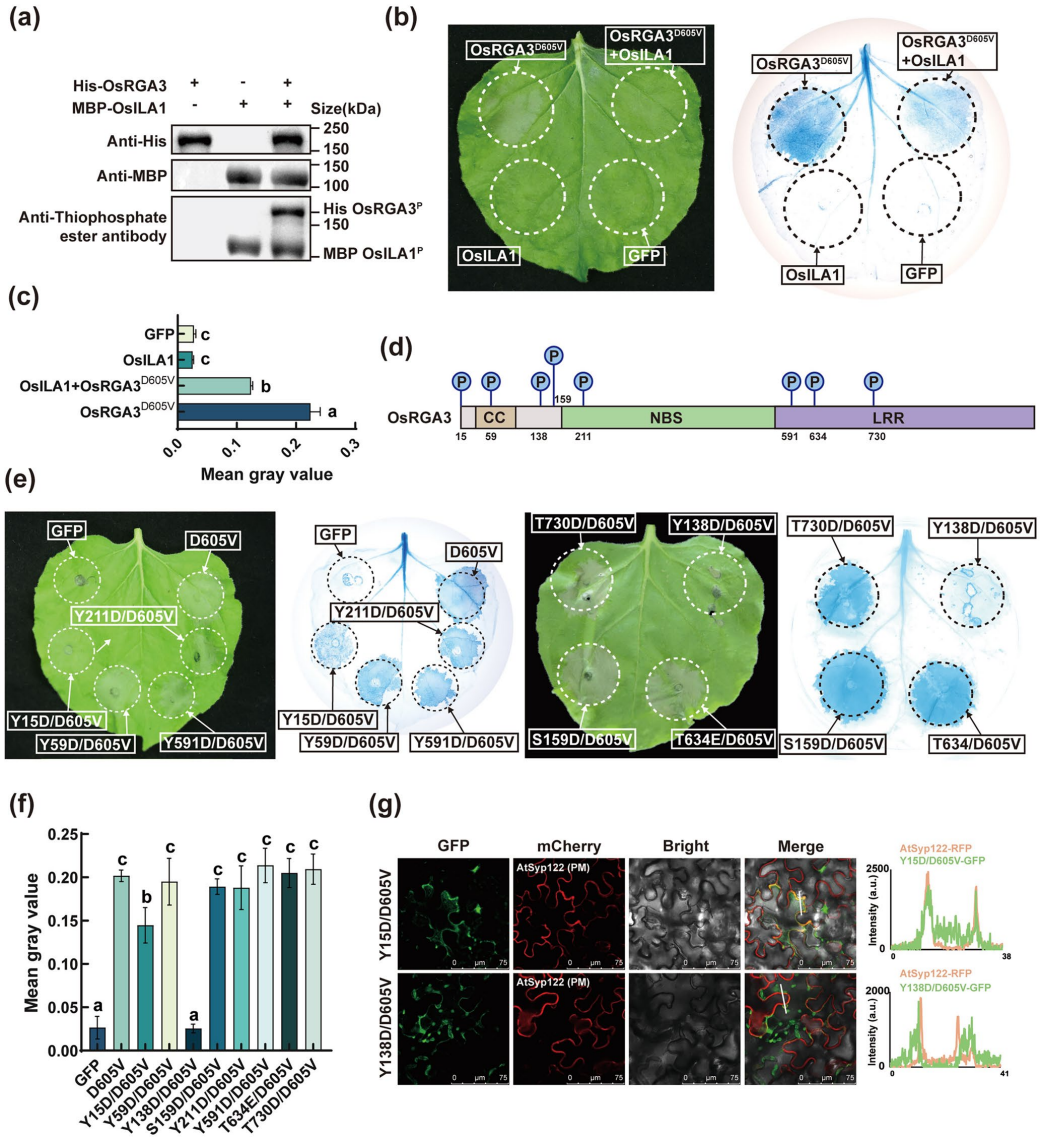
OsILA1 inhibits the function of OsRGA3^D605V^ by phosphorylating the Y15 and Y138 sites. (a) The result of the immunoblot analysis after OsRGA3 is phosphorylated by OsILA1. (b) OsRGA3^D605V^‐GFP with OsILA1‐GFP was expressed transiently in *Nb* leaves and stained with trypan blue. (c) The grey value statistical results involved in (g). (d) Schematic diagram of eight candidate phosphorylation sites in OsRGA3 after phosphorylated by OsILA1 in vitro. (e) The eight candidate sites were mutated separately in OsRGA3^D605V^ and expressed in *Nb*. (f) The grey value statistical results involved in (e). (g) Confocal microscopy was utilised to examine GFP‐OsRGA3^D605V/Y15D^ and GFP‐OsRGA3^D605V/Y138D^ in *Nb* leaves. Bars, 75 μm. Error bars in (c) and (f) indicate ± SD (*n* = 3). The *p* values were determined by one‐way ANOVA with two‐sided Tukey's HSD test and indicated with different letters, *p* < 0.01.

As a Raf‐like MAPKKK, OsILA1 can phosphorylate OsRGA3 (Figure 6a), and inhibits HR induced by OsRGA3^D605V^ (Figure 6b,c). Furthermore, we conducted mass spectrometry analysis of OsRGA3 phosphorylated by OsILA1 in vitro and identified eight candidate phosphorylation sites (Figure 6d). These eight sites were mutated individually in OsRGA3^D605V^ and expressed in *Nb*, respectively. The results showed that, compared with OsRGA3^D605V^, the grey value of HR induced by OsRGA3^D605V/Y15D^ is significantly reduced, and OsRGA3^D605V/Y138D^ resulted in the loss of HR (Figure 6e,f). At the same time, the concentration of calcium ions in *Nb* tissues after OsRGA3^D605V/Y15D^ and OsRGA3^D605V/Y138D^ expression was significantly lower than that of OsRGA3^D605V^ (Figure S15b). Further subcellular localisation experiments showed that mutants of OsRGA3^D605V/Y15D^ and OsRGA3^D605V/Y138D^ exhibited reduced PM localisation (Figure 6g). These results indicated that OsILA1 through phosphorylated OsRGA3, affected its function by altering its localisation, thereby avoiding excessive immune response that could negatively impact plant growth.

## 3 Discussion

**FIGURE 7:**
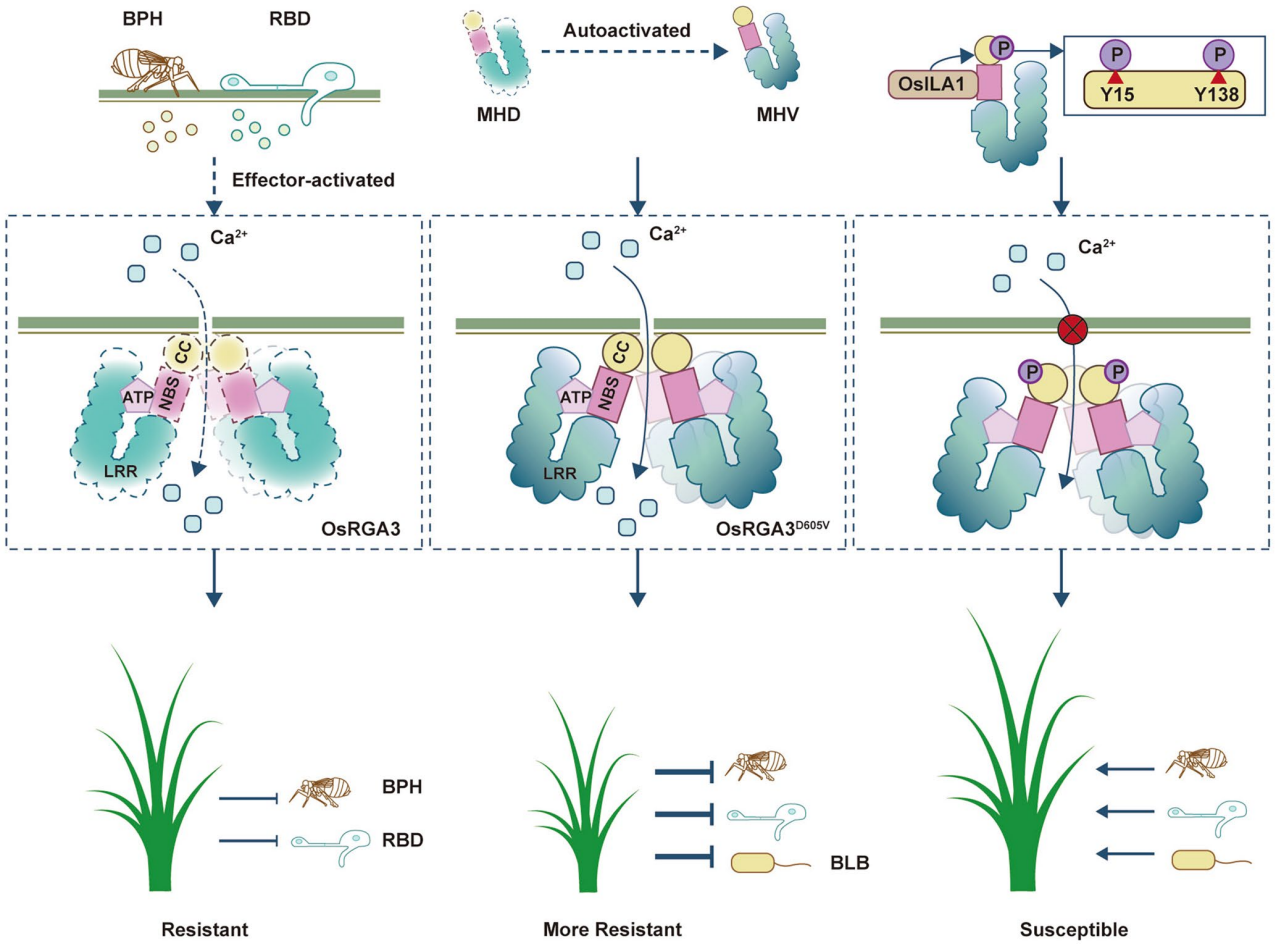
Proposed model for the OsRGA3‐regulated rice resistance to BPH and RBD. When rice is attacked by BPH and RBD, the effectors released by the pathogens and insects activate the NLR OsRGA3, causing it to oligomerize on the PM through CC domain, inducing calcium ion influx, thereby activating the rice immune response and enhancing rice resistance to BPH and RBD. By modifying the MHD motif in OsRGA3 to MHV, the plant tissue's HR can be automatically activated without the need for effector activation, conferring stronger resistance to rice against BPH, RBD, and BLB but affecting rice growth. The Raf‐like MAPKKK OsILA1 interacts and with the NBS domain of OsRGA3, phosphorylating the CC domain of OsRGA3, inhibiting PM localisation of OsRGA3 and preventing cell death caused by calcium ion influx, thereby attenuating the resistance of rice to biotic stresses and sustaining growth. This process plays a role in the arms race between rice and pathogens.

As a significant threat to the growth and development of rice, BPH infests rice by ingesting phloem sap through its stylet mouthpart (Zhang et al. 2019), and RBD invades rice cells by appressoria penetration of the cell walls (Dean et al. 2012), both employing similar invasion strategies. Previous studies have found that the knockout of the gene *OsF3'H* enhances rice resistance to both BPH and RBD (Chen et al. 2022). In this study, we discovered that the NLR proteins of OsRGA3 and OsRGA3^D605V^ positively regulate rice resistance to multiple biotic stresses and identify a new MAPK OsILA1‐NLR module that can balance rice growth and resistance (Figure 7), providing new insight for the breeding of high‐yielding rice with broad‐spectrum resistance.

Multiple studies have demonstrated that substituting histidine or aspartic acid within the MHD motif with other amino acids results in auto‐activation of NLR proteins (Bendahmane et al. 2002; Gao et al. 2011; Howles et al. 2005; Van Bentem et al. 2005). We observed OsRGA3^D605V^ leads to a robust HR in tobacco *Nb* and endows rice with stronger and enhanced broad‐spectrum resistance compared with OsRGA3 (Figure 2). As the autoactivated form of OsRGA3, OsRGA3^D605V^ may exhibit a more rapid immune response by skipping the process of pathogen recognition and activation. The mechanism by which OsRGA3 recognises pathogens and the potential for other NLRs to function synergistically with OsRGA3 in modulating plant immunity requires further investigation.

Upon effector recognition, NLRs oligomerize through the CC domains to form a resistosome (Förderer et al. 2022; Ma et al. 2020; Martin et al. 2020; Wang et al. 2019; Zhao et al. 2022), initiating calcium ion influx by forming Ca^2+^ permeable channels. As reported for the *Arabidopsis* NLRs AtRPM1 and ZAR1, both of which lead to a surge in Ca^2+^ in cytosolic and HR, with LaCl_3_ application inhibiting the HR induced by AtRPM1 (Bi et al. 2021; Grant et al. 2000). We found that OsRGA3^D605V^ exhibited PM localisation (Figure 3a,b), and the CC domains of OsRGA3 interact with each other, indicating that OsRGA3 can form a resistosome (Figure 3c). The induction of HR by OsRGA3^D605V^ suppressed by LaCl_3_, resulted in significantly higher tissue Ca^2+^ concentrations compared to the control (Figure 3d,e and Figure S6). Moreover, we observed that after BPH infection, the Ca^2+^ concentrations were significantly elevated in the tissues of OsRGA3‐OE2, osila1, and OsRGA3^D605V^‐OE rice lines, and exogenous LaCl_3_ suppressed BPH resistance in OsRGA3‐OE plants. This suggests that the HR triggered by OsRGA3^D605V^ is due to Ca^2+^ influx, and it is probable that OsRGA3^D605V^ forms a resistosome and functions as a Ca^2+^ permeable channel in the PM.

The CC domain of NLR proteins, particularly the α1 helix, serves as a critical switch for the membrane localisation and immune function of NLRs (Bi et al. 2021; Gao et al. 2020; Wang et al. 2019). We found in BPH‐susceptible rice varieties that the OsRGA3 predominantly lacks the N‐terminal CC domain, and the protein segment missing the CC domain fails to properly localise to the membrane (Figure S7b and Figure 4d), illustrating the pivotal role of the CC domain in protein PM targeting and function. Additionally, in the BPH‐resistant rice variety RHT, OsRGA3 lacks the C‐terminal LRR domain, which has lower conservation and inhibits NLR function in a quiescent state (Figure S7b). The absence of LRR leads to the exposure of ATP binding sites within RHT OsRGA3 (Figure S7c), which may result in a more rapid ADP‐ATP exchange, and thus a more rapid response to pathogen invasion. Moreover, compared with japonica, indica accessions more frequently harbour the same OsRGA3 genotype present in RHT and display stronger BPH resistance. Above all, the protein domain deletion polymorphism of OsRGA3 in various rice varieties may lead to different rice resistance responses and the different domains of OsRGA3 may influence each other through collaboration or antagonism.

Studies have indicated that the fragments of NLRs are sufficient to induce HR. For instance, the N‐terminal fragments of wheat NLR MLA10, Sr33, and Sr50 can trigger HR in *Nb* (Cesari et al. 2016). The eCC domain of the wheat powdery mildew resistance NLR Pm21 can interact with itself to induce HR, whereas the full‐length Pm21 cannot (Gao et al. 2020). There are also the fragments of NLRs that fail to induce HR; for example, AtRPM1 does not exhibit an activating function even with the introduction of auto‐activating MHD mutations into the fragments (El Kasmi et al. 2017). In this study, we divided OsRGA3 into eight distinct fragments based on its domains which failed to induce HR, and the fragments of OsRGA3^D605V^ also could not induce HR (Figure 4a–c). Specifically, OsRGA3^CNL‐DV^, compared to OsRGA3^D605V^, lacked only the first 20 N‐terminal amino acids and still localised to PM (Figure 4d). This suggests that the absence of the first 20 N‐terminal amino acids in OsRGA3 does not affect the PM localisation but impacts plant immune responses.

The MAPK pathway serves as a pivotal downstream signalling transduction route for Pattern Recognition Receptors (PRRs) (Asai et al. 2002; Gao et al. 2008), which can also regulate plant immunity in ETI. For instance, the disruption of the MEKK1‐MKK1/MKK2‐MPK4 kinase cascade results in the activation of NLR SUMM2‐mediated immunity (Zhang et al. 2012). Our research revealed that OsILA1 phosphorylated OsRGA3 and suppressed the HR caused by the mutant OsRGA3^D605V^ in *Nb*, negatively regulating the resistance of rice (Figures 5 and 6). Notably, the OsRGA3^CNL‐DV^ lacking the first 20 N‐terminal amino acids did not induce HR (Figure 4), and phosphorylation at the Y15 residue attenuated the HR caused by OsRGA3^D605V^ (Figure 6e), indicating that the Y15 residue is likely a critical site for HR induction by OsRGA3^D605V^. Additionally, unlike the ZH11, NIP, and RHT, the amino acid alignment of OsRGA3 shows that at position 138, histidine is present in the BPH‐susceptible rice varieties Kitaake, 9311, MH63, and ZS97, with the N‐terminus of OsRGA3 in Kitaake not being truncated (Figure S7b). We found that phosphorylation at the Y138 residue directly resulted in the disappearance of HR (Figure 6e), implying that mutations at Y138 affected the function of OsRGA3, leading to the loss of activity in some rice varieties and consequently increasing their sensitivity to biotic stress.

When confronted with pathogens, plants allocate more nutrients for immune pathways; hence NLRs may potentially impact plant growth and development while enhancing plant immunity (Chakraborty et al. 2018; Deng and He 2024; Karasov et al. 2017; Qi et al. 2021; Sun et al. 2023). OsRGA3 positively regulates rice resistance to BPH and RBD without significantly affecting normal growth (Figure S4), but OsRGA3^D605V^‐OEs show shorter tillering‐stage height and panicle seed setting rate. This reflects the growth penalty arising from auto‐activated immunity conferred by OsRGA3^D605V^. Besides, overexpression of OsILA1 significantly increased growth and decreased 1000‐grain weight. This may relate to genes in growth‐promoting pathways directly regulated by *OsILA1*, diverting more carbon to vegetative growth and less grain filling; the underlying mechanism awaits further investigation.

In conclusion, our results describe a signalling pathway that involves OsILA1 and OsRGA3 mediated resistance to BPH and RBD, providing new insights into the molecular mechanisms underlying rice resistance to BPH, RBD and BLB, as well as avenues for the development of broad‐spectrum resistant rice cultivars.

## 4 Material and Method

### 4.1 Plant and Insect Materials

ZH11 ( *Oryza sativa* L. subsp. japonica cv Zhonghua no. 11, ZH11) was used as the wild‐type rice in this study. Rice is cultivated in a greenhouse at 26°C ± 2°C, with a light:dark cycle of 12 h:12 h, and a relative humidity of 70%–80%, or planted in the summer at a farm in Songjiang, Shanghai, China.

BPH was initially collected from rice fields in Shanghai and maintained on BPH‐susceptible rice cultivar Taichung Native 1 (TN1) seedlings in a climate‐controlled room at 26°C ± 2°C, with a light:dark cycle of 12 h:12 h, and a relative humidity of 70%–80%.

### 4.2 Plasmid Construction and Plant Transformation

All primers utilised in this study are detailed in Table S1.

The sgRNA for OsRGA3 was designed using the web resource http://skl.scau.edu.cn, and the CRISPR (Clustered regularly interspaced short palindromic repeats)/Cas9 system was assembled following the established protocol (Ma et al. 2015).

All recombinant plasmids were introduced into ZH11 via a modified *Agrobacterium tumefaciens* ‐mediated transformation technique (Hiei et al. 1994).

### 4.3 BPH Resistance Detection and Analysis

Rice resistance to BPH was assessed in individual plants and small populations using the previously described methods (Du et al. 2009; Guo et al. 2014, 2020).

To evaluate individual plants, single rice plants were grown in 7 cm^2^ pots, covered with transparent plastic cylinders (6 cm in diameter; 30 cm in height), and at the three‐leaf stage, 15 s‐ to third‐instar BPH nymphs were added to each plant, with six replicates for each material.

To evaluate small populations of plants, 40 rice plants per line were grown in plastic pots (length × width × height = 30 × 20 × 8.5 cm), and after 3 weeks, each plant was inoculated with five second‐ to third‐instar nymphs of BPH. The condition of the plants was checked daily until most seedlings of the various lines wilted, after which the seedling mortality rate was recorded. Each material was replicated three times.

### 4.4 Plant Inoculation Experiments

The rice blast pathogen ( *M. oryzae* TH12) and BLB (*Xanthomonas oryzae pv. oryzae*, *XOO*) strain PXO99 were kindly provided by Prof. Zuhua He (CAS Center for Excellence in Molecular Plant Sciences).

The modified in vitro leaf inoculation method previously described was used to inoculate rice leaves with RBD (Chen et al. 2022; Deng et al. 2017; Zhai et al. 2019). TH12 was cultured on full‐nutrient agar for two weeks, and a spore suspension (1 × 10^5^ spores mL^−1^) was prepared and used within 2 h. One month after transplanting, leaf segments of 5–6 cm were cut from 5 cm below the tip of the second leaf from the top of the rice, and TH12 spore suspension (5 μL) was inoculated through a wound. The leaves were then placed in a growth chamber (temperature at 28°C, with a light:dark cycle of 12 h:12 h).

The typical symptom of RBD manifests as a spindle‐shaped lesion with a grey center, surrounded by brown necrotic tissue, and an outer layer of light yellow. The area of the lesions was measured on the seventh day post‐inoculation using a ruler and the ImageJ software to determine the resistance differences of rice varieties to TH12. Each material was replicated more than 30 times.

To measure fungal growth, the copy number of *MoPot2* in TH12 on the leaves was quantified using a modified published DNA‐Based Real‐Time PCR method (Kawano et al. 2010), with the rice gene *OsUBQ* serving as a reference standard, and each material was replicated in triplicate, and data from three biological replicates were collected; the mean values with standard deviation were plotted.

To measure BLB resistance, rice flag leaves were inoculated with BLB using the leaf‐clipping method at the booting stage, which has been previously detailed (Sun et al. 2004). The disease symptoms were evaluated by determining the relative lesion length 14 days post‐inoculation.

### 4.5 Quantitative Real‐Time Polymerase Chain Reaction (qRT‐PCR)

Total RNA was extracted using TRIzol (Invitrogen). The RNA was reverse transcribed into cDNA using the ReverTra Ace qPCR RT Master Mix with gDNA Remover (Toyobo). The qRT‐PCR analysis was performed using the SYBR Green Real‐time PCR Master Mix Kit (Toyobo), with *OsActin* and *OsUBQ* selected as reference genes. Each sample was analyzed in triplicate, and data from three biological replicates were collected; the mean values with standard deviation were plotted.

### 4.6 Amino Acid Sequence Analysis and Three‐Dimensional Structure Prediction

Amino acid sequences were downloaded from NCBI (www.ncbi.nlm.nih.gov/). The Clustal W algorithm in MEGA 11 was used to compare different amino acid sequences and perform homology analysis, and a phylogenetic tree was constructed using the neighbour‐joining method. The results of the amino acid sequence alignment were visualised in GENEDOC. The three‐dimensional structure of the OsRGA3 protein was retrieved from the AlphaFold Protein Structure Database (ebi.ac.uk), and the three‐dimensional structures of the mutated protein and other protein fragments were predicted using AlphaFold2.

### 4.7 Trypan Blue Staining

Using the previously described staining method with slight modifications (Bai et al. 2012), *Nicotiana benthamiana* (*Nb*) leaves were immersed in a solution of trypan blue dye (10 mL lactic acid, 10 mL glycerol, 10 g phenol, and 10 mg trypan blue, dissolved in 10 mL of ddH_2_O). The leaves with dye were boiled for 5 min, left at room temperature for 1 h, and then rinsed in a saturated solution of chloral hydrate for 3 days before observing the results. The mean grey value was measured using ImageJ.

### 4.8 Membrane Fractionation Assays

Plasma‐membrane (PM) proteins were enriched by slightly modifying a previously described protocol (Jacob et al. 2021). Homogenised tissue was extracted in sucrose buffer (20 mM Tris–HCl pH 8.0, 0.33 M sucrose, 1 mM EDTA, 5 mM DTT, 1× plant protease inhibitor), cleared at 2000 *g*, and the total protein (T) was re‐spun at 20 000 *g* to yield cytoplasmic fraction (C). The pellet was resuspended in kit buffer B (Minute PM protein isolation kit, Invent Biotechnologies), layered onto PBS, and centrifuged at 16 000 *g* to collect the PM fraction. Samples were analysed by SDS‐PAGE and western blot.

### 4.9 Ca^2+^ Concentration Detection

The Ca^2+^ concentration in *Nb* leaves after 72 h of protein expression and in rice stems weredetected according to the manufacturer's instructions (S1063S; Beyotime Biotechnology, Shanghai, China), and chemiluminescence was measured on a full‐spectrum multifunctional microplate reader (Varioskan Flush).

### 4.10 Yeast Two‐Hybrid (Y2H) Assay

For the Y2H assay, the coding sequences (CDSs) of the target gene were constructed into the pGADT7/pGBKT7 vectors (Clontech, Mountain View, CA, USA), and protein–protein interactions were detected according to the manufacturer's instructions (YC1002; Weidi Biotechnology, Shanghai, China).

### 4.11 Luciferase Complementation Assay (LCA) and Bimolecular Fluorescence Complementation (BiFC) Assay

For LCA, the coding sequences (CDSs) of the target gene were constructed into the pCAMBIA‐35S‐nLuc and pCAMBIA‐35S‐cLuc vectors. For the BiFC assay, the tested CDSs were amplified by PCR and inserted into pCAMBIA1300‐35S‐nYFP and pCAMBIA1300‐35S‐cYFP.

All recombinant plasmids were inserted into *A. tumefaciens* strain GV3101 (pSoup‐p19), which was transformed into *Nb* leaves with infiltration buffer (10 mM MgCl_2_, 150 μM Acetosyringone, and 10 mM MES at pH 5.6), with the suspension having an OD_600_ of 1. At 48 h post infiltration, a fluorescein solution at a concentration of 150 μg/mL was injected, and the LUC signal was detected using a CCD camera. Confocal microscopy (Leica TSC SP8 STED 3X) was used to detect the YFP signal.

### 4.12 MBP Pull‐Down

For the in vitro MBP pull‐down assay, the CDS sequences of OsILA1 and OsRGA3 were individually cloned into the pMAL‐c5X and pCold‐TF vectors, which expressed as MBP‐OsILA1 and His‐OsRGA3 in the BL21 (DE3) strain. The proteins were purified using amylose resin (E8021; NEB, Ipswich, MA, USA) and Ni‐NTA resin (L00250; GenScript, Nanjing, China). Next, 6 μg of MBP‐OsILA1 and 6 μg of His‐OsRGA3 were incubated with 25 μL of amylose resin at 4°C for 2 h in 200 μL binding buffer (150 mM NaCl, 0.2% Triton X‐100, 10% glycerol, and 0.5 mM PMSF). The amylose resin was washed five times with wash buffer (500 mM Tris–HCl, pH 8.0, 140 mM NaCl, 0.1% TritonX‐100, 1 mM EDTA) and then the precipitates were boiled in 1× SDS loading buffer and detected by immunoblotting using the corresponding antibodies (E8032S; NEB, and 30401ES50; Yeasen, Shanghai, China).

### 4.13 BLI Assay

Purified His‐OsILA1 was biotinylated and loaded onto a Ni‐NTA biosensor until a baseline shift of ~1 nm was reached. After a 60‐s buffer equilibration, the sensor was dipped into a running buffer (PBS, 0.02% Tween‐20) containing a two‐fold serial dilution series of His‐OsRGA3. Association was monitored for 300 s, followed by 600 s of dissociation in fresh buffer. Sensorgrams were double‐referenced, and a 1:1 Langmuir binding model was applied to calculate the equilibrium dissociation constant (K_D_).

### 4.14 In Vitro Phosphorylation Assays

For the in vitro phosphorylation reaction, 4 μg of the target protein His‐OsRGA3, 8 μg of MBP‐OsILA1, and 400 μM ATP were mixed, and the kinase reaction buffer (25 mM Tris–HCl, 10 mM MgCl2, 1 mM DTT) was added to a final volume of 30 μL. The reaction was carried out at 25°C for 45 min, after which protein electrophoresis was performed for mass spectrometry analysis.

For the immunoblot analysis, 4 μg of the target protein His‐OsRGA3, 8 μg of MBP‐OsILA1, and 200 μM ATP‐γ‐S were mixed, and the kinase reaction buffer was added to a final volume of 30 μL. The reaction was carried out at 25°C for 30 min, after which 50 mM PNBM (p‐Nitrobenzyl mesylate) was added and the reaction continued for an additional 45 min. The immunoblot analysis was performed using a rabbit anti‐thiophosphate ester antibody (ab92570; Abcam).

### 4.15 Subcellular Localisation

The plasmid pCAMBIA1300‐35S‐AtSyp122‐mCherry, provided by Prof. Feng Zhou (CAS Center for Excellence in Molecular Plant Sciences), was used as a marker for PM localisation. The CDSs of the indicated genes were amplified by PCR and inserted into pCAMBIA1301‐35 s‐eGFP. The proteins fused with eGFP were transiently expressed in *Nb* as described above. After 48 h, the samples were observed using confocal microscopy (Leica TSC SP8 STED 3X). Fluorescence intensity was compared using ImageJ software.

### 4.16 Statistical Analysis

The two‐tailed unpaired Student's *t*‐test and one‐way ANOVA with two‐sided Tukey's HSD test were used to compare the difference in mean ± SD and a *p*‐value of ≤ 0.05 was considered significant for the assays about qRT‐PCR, the small population, lesion area, DNA‐Based Real‐Time PCR, mean grey value and calcium ion concentration. The exact value of the sample size (*n*) is represented in the figure or legend.

### 4.17 Accession Numbers of the Genes Used in This Study

Sequence data for the genes described herein is available in the China Rice Data Center databases under the following accession nos.: *OsRGA3, LOC_Os11g11770; OsILA1, LOC_Os06g50920; OsPR5, LOC_Os12g43380; OsPR10a, LOC_Os12g36880; OsJAZ8, LOC_Os09g26780; OsUBQ, LOC_Os03g13170; OsActin, LOC_Os03g50885; BPH14, LOC_Os03g63150; BPH30, LOC_Os04g02520; BPH6, LOC_Os04g35210; BPH1/9, LOC_Os12g37280*.

## Author Contributions

Y.Z. performed the experiments; X.M. and S.C. conceived and supervised the project; X.M. and H.L. designed the experiments. B.S., H.L., Z.S. and X.M. discussed results and offered advice. Y.Z., M.L. and H.L. analysed the data. Y.Z., X.M. and H.L. wrote the manuscript; X.M., B.S. and H.L. revised the manuscript.

## Funding

This study was supported by grants from the Shanghai Chongming Agricultural S&T Innovation Project, China (Grant No. 2023CNKC‐01‐04).

## Conflicts of Interest

The authors declare no conflicts of interest.

## Supporting information


**Appendix S1:** pbi70471‐sup‐0001‐supinfo.docx.

## Data Availability

The data that support the findings of this study are available on request from the corresponding author. The data are not publicly available due to privacy or ethical restrictions.
