# Supplementary material for: An Auto‐Activated NLR‐Protein OsRGA3D605V Confers Rice Triple Resistance and Deactivates Resistance After Phosphorylation by OsILA1
Source: Plant Biotechnol J. 2025 Nov 27;24(4):2126–42. doi: 10.1111/pbi.70471 (PMC13140720; doi:10.1111/pbi.70471)
Supplement: Supplementary file 1 — Appendix S1: pbi70471‐sup‐0001‐supinfo.docx. [file PBI-24-2126-s001.docx]

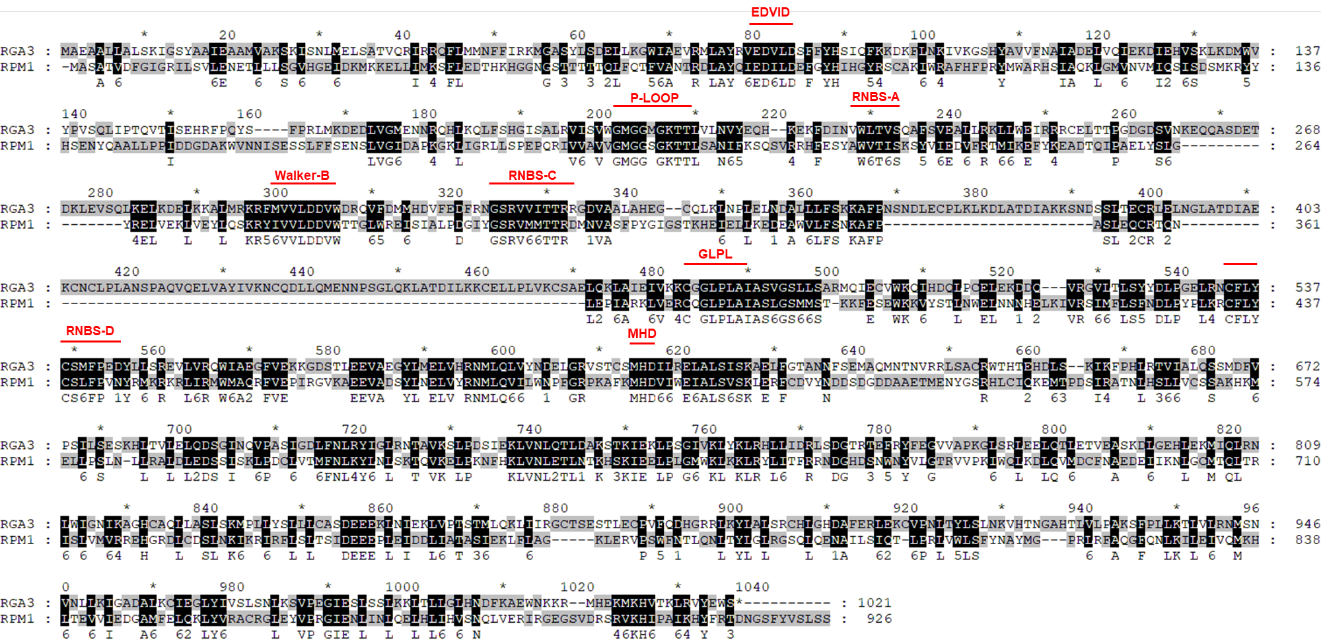


**Fig. S1 Amino acid sequence alignment for OsRGA3 and AtRPM1, with a focus on conserved structural domains.**

**
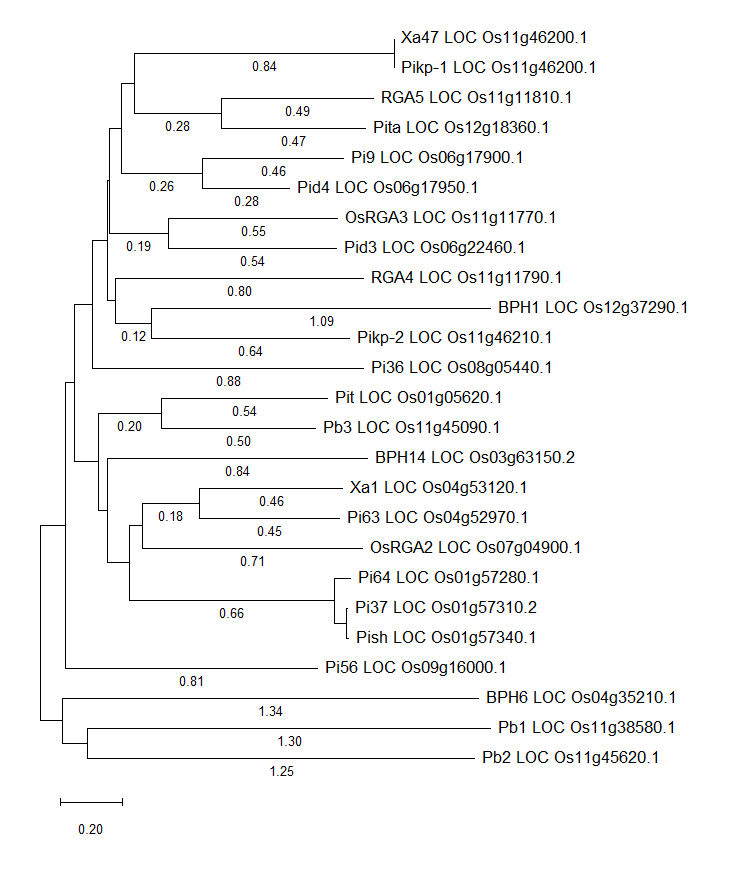
**

**Fig. S2 Evolutionary and developmental analysis of the rice NLR family proteins.**

The phylogenetic tree was generated using the full-length amino acid sequences of the cloned rice NLR family members according to the Neighbor-Joining method in MEGA7.0. The numerical values represent the branch lengths, which are hidden if shorter than 0.05.


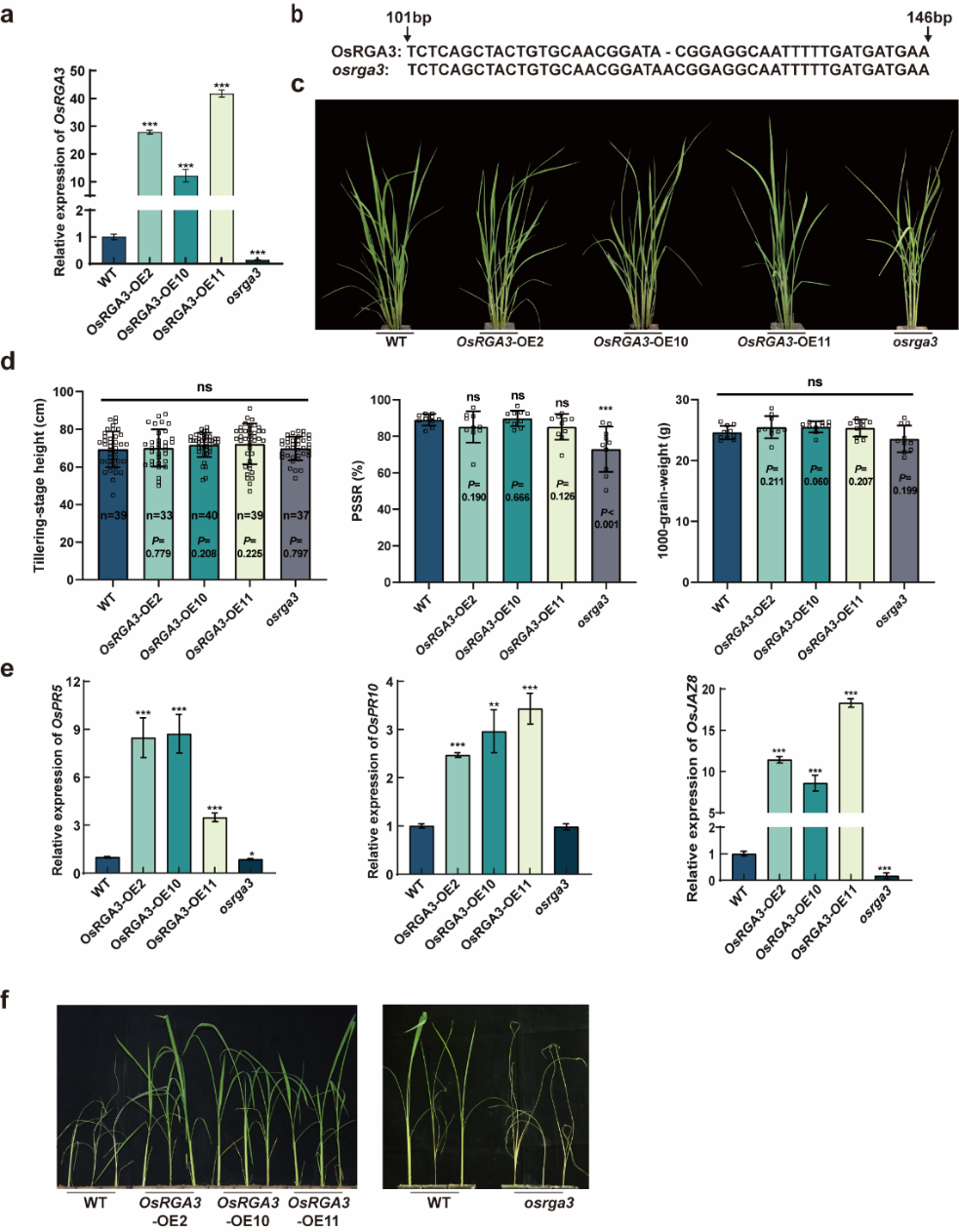


**Fig. S3 Construction and detection of *OsRGA3* transgenic lines.**

**a** Results of the qRT-PCR analysis of *OsRGA3* transcription in *OsRGA3* transgenic lines. **b** Schematic diagram of the knockout of the *OsRGA3* gene locus in *osrga3*, insertion of an adenine base at the nucleotide position between the 123rd and 124th base pairs within the coding sequence (CDS). **c** The tillering stage of *OsRGA3* transgenic lines in the field. **d** Statistical results of tillering-stage height, panicle seed setting rate (PSSR, n =10), and 1000-grain-weight (n =10) of field-grown plants. **e** Results of the qRT-PCR analysis of *OsPR5, OsPR10* and *OsJAZ8* transcription in OsRGA3 transgenic lines, n =3. **f** The plant status of OsRGA3 transgenic lines after BPH infestation in single plant test. Error bars in **(a)**, **(d)** and **(e)** indicate ± SD. The *p* values were determined by two-tailed unpaired Student’s *t*-test, ^∗^*p* < 0.05, ^∗∗^*p* < 0.01, ^∗∗∗^*p* <0.001.


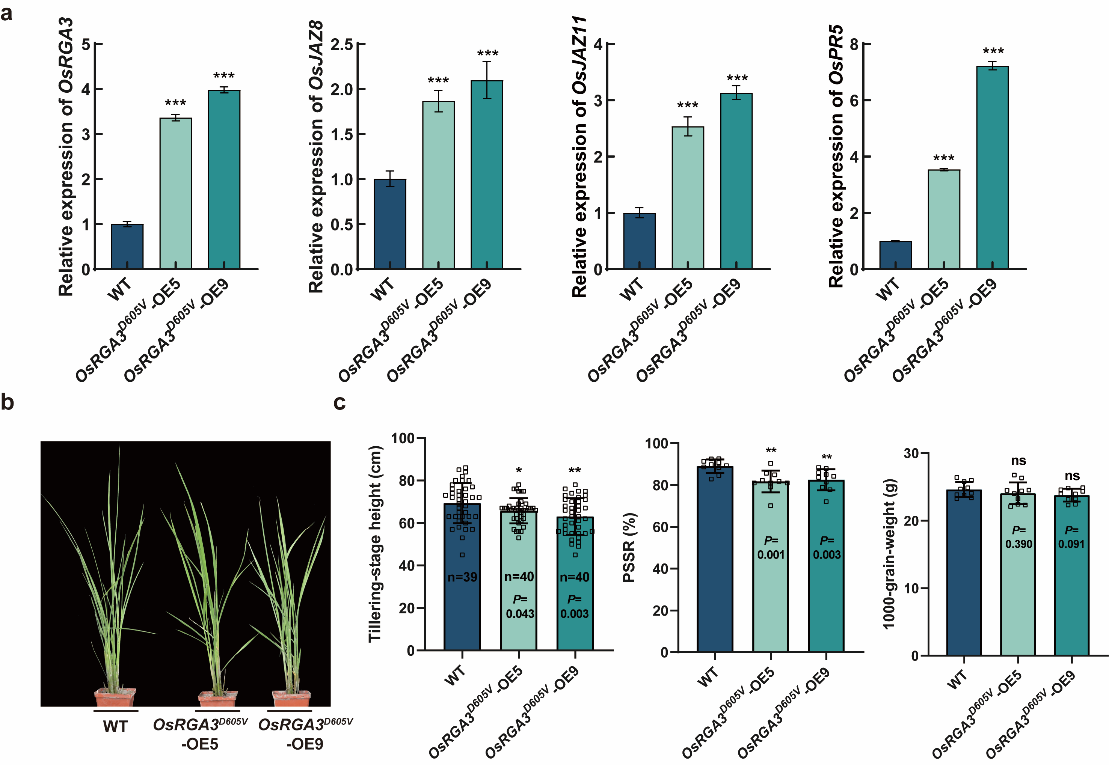


**Fig. S4 OsRGA3^D605V^ confers resistance to BPH in rice.**

**a** Results of the qRT-PCR analysis of *OsRGA3*, *OsJAZ8, OsJAZ11* and *OsPR5* transcription in transgenic rice overexpressing OsRGA3^D605V^. b The tillering-stage of *OsRGA3^D605V^* transgenic lines in the field. c Statistical results of tillering-stage height, panicle seed setting rate (n =10), and 1000-grain-weight (n =10) of field-grown plants. Error bars in **(a)** and **(c)** indicate ± SD. The *p* values were determined by two-tailed unpaired Student’s t-test, *^∗^p* < 0.05, *^∗∗^p* < 0.01, *^***^p* < 0.01.


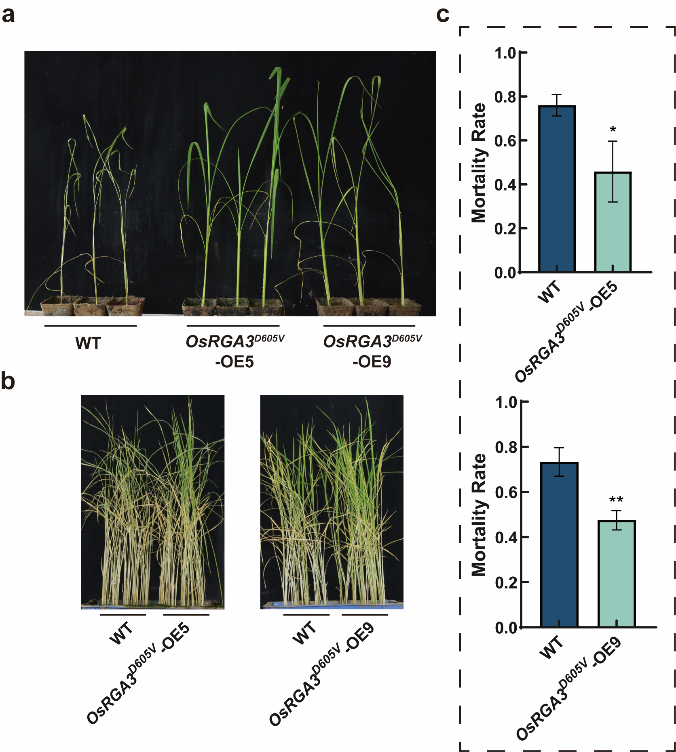


**Fig. S5 OsRGA3^D605V^ confers resistance to BPH in rice.**

The plant status **(a, b)** and the survival rate **(c)** of *OsRGA3^D605V^* transgenic lines after BPH infestation in single plant **(a)** and small population tests **(b)**. Error bars in **(c)** indicate ± SD (n = 3).The *p* values in **(c)** were determined by two-tailed unpaired Student’s *t*-test, ^∗^*p* < 0.05, ^∗∗^*p* < 0.01.


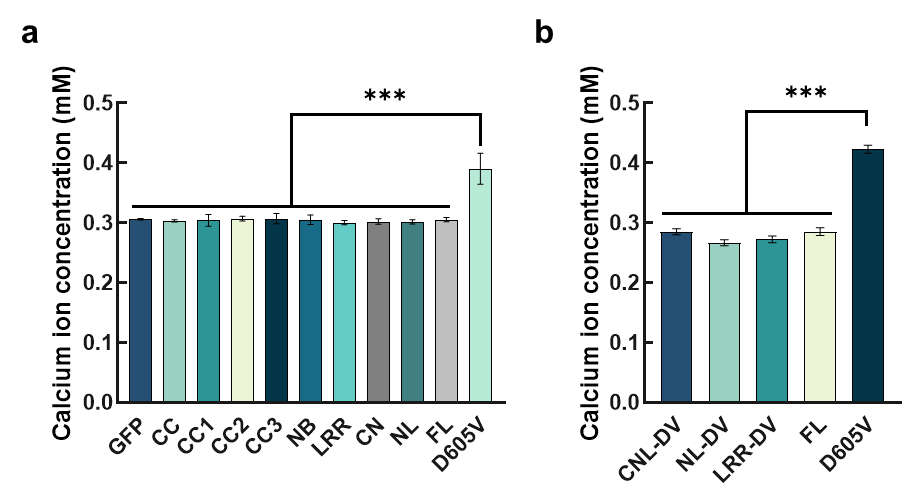


**Fig. S6 Ca^2+^ concentration detection in *Nb* leaves**

**a, b** The calcium ion concentration in *Nb* leaves 72 hours after the different protein fragments expression. Error bars **(a)** and **(b)** indicate ± SD (n = 3). The *p* values were determined by two-tailed unpaired Student’s *t*-test, ^∗∗∗^*p* < 0.001.


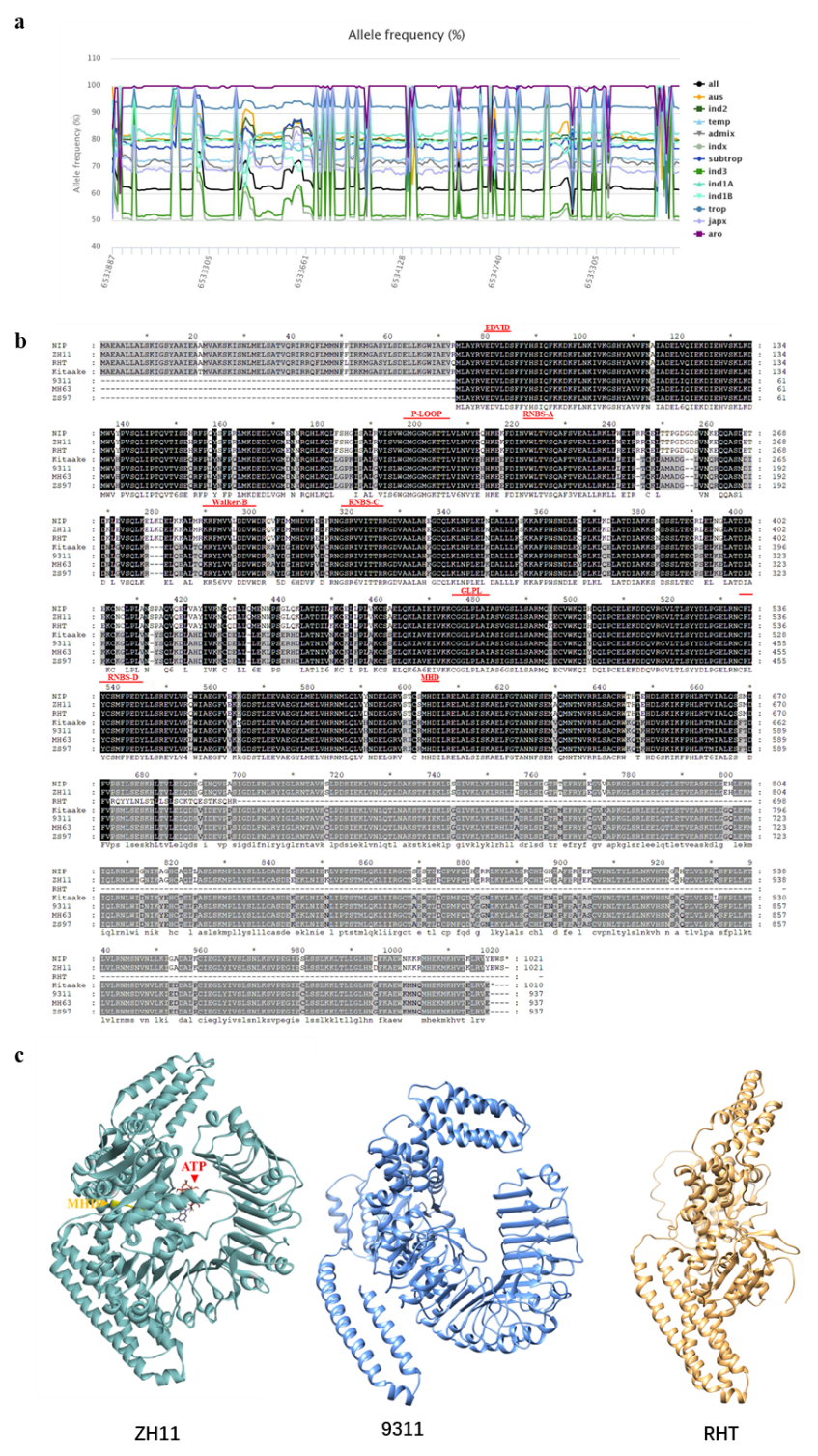


**Fig. S7 Structural analysis of the OsRGA3 protein**

**a** Online analysis of the *OsRGA3* gene sequences within the genomes of different rice varieties (https://snp-seek.irri.org/_snp.zul). **b** Alignment results of the amino acid sequences encoded by the *OsRGA3* in seven different rice varieties. The red line indicates a conserved motif. **c** Prediction of the three-dimensional structure of the OsRGA3 protein encoded in rice varieties ZH11, 9311, and RHT. The yellow arrow indicates the MHD motif, and the red arrow points to the predicted ATP-binding site.

**Fig. S8 Distribution of *OsRGA3* haplotypes carrying the resistance-linked 1-bp deletion across 3,000 rice accessions.**


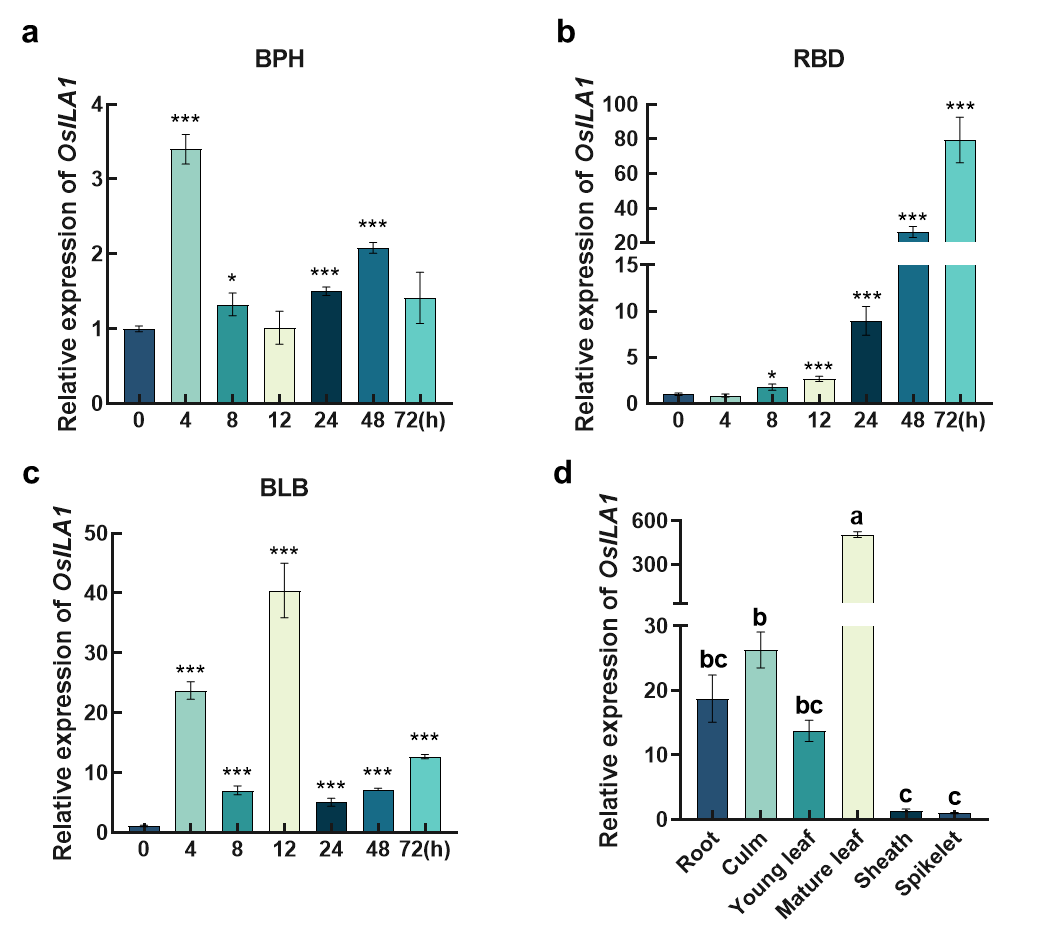


**Fig. S9 Analysis of the expression pattern of *OsILA1***

**a-c** Result of the qRT-PCR analysis of *OsILA1* transcription in rice during the BPH infestation **(a)**, the RBD **(b)** and BLB infection **(c)**. **d** Result of the qRT-PCR analysis of *OsILA1* transcription in different rice tissues. Error bars in (a-d) indicate ± SD (n = 3). The *p* values in **(a-c)** were determined by two-tailed unpaired Student’s *t*-test, ^∗^*p* < 0.05, ^∗∗∗^*p* < 0.001. The *p* values in **(d)** were determined by one-way ANOVA with two-sided Tukey’s HSD test and indicated with different letters, *p* <0.01.


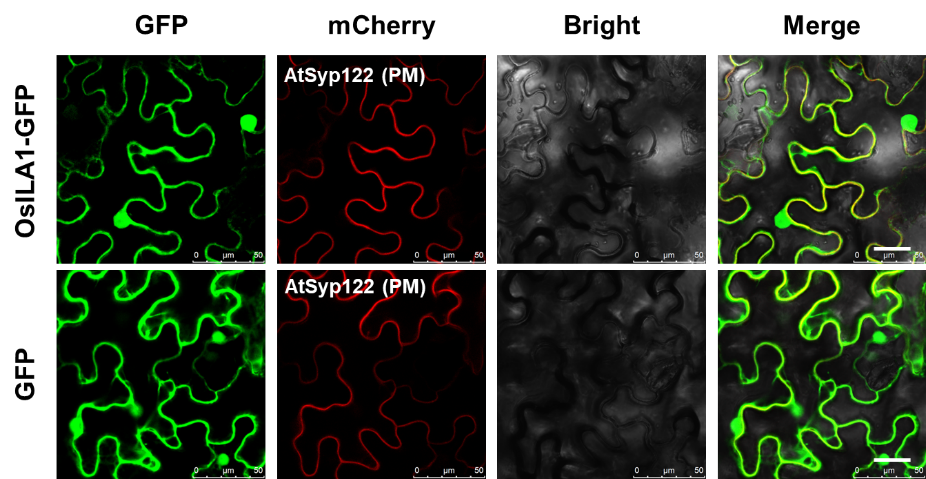


**Fig. S10 Subcellular localization of OsILA1**

Confocal microscopy was utilized to examine the OsILA1 fused with a C-terminal GFP. Bars, 25 µm.

**Fig. S11** **BLI assays demonstrate a direct interaction between OsRGA3 and OsILA1.**


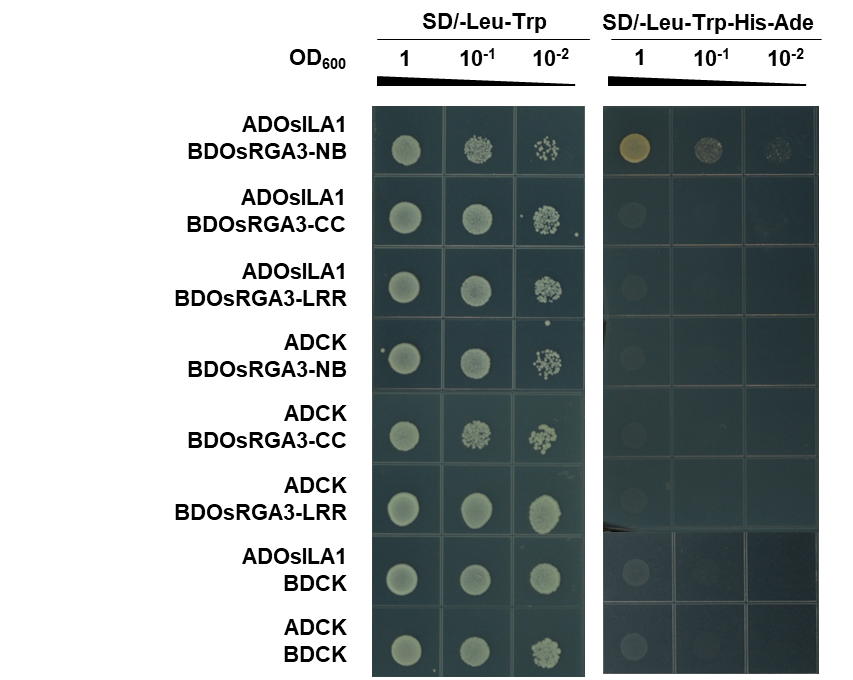


**Fig. S12 OsILA1 interacts with the NBS domains of OsRGA3.**

Y2H assay results for the interaction between OsILA1 with the different domains in OsRGA3.

**
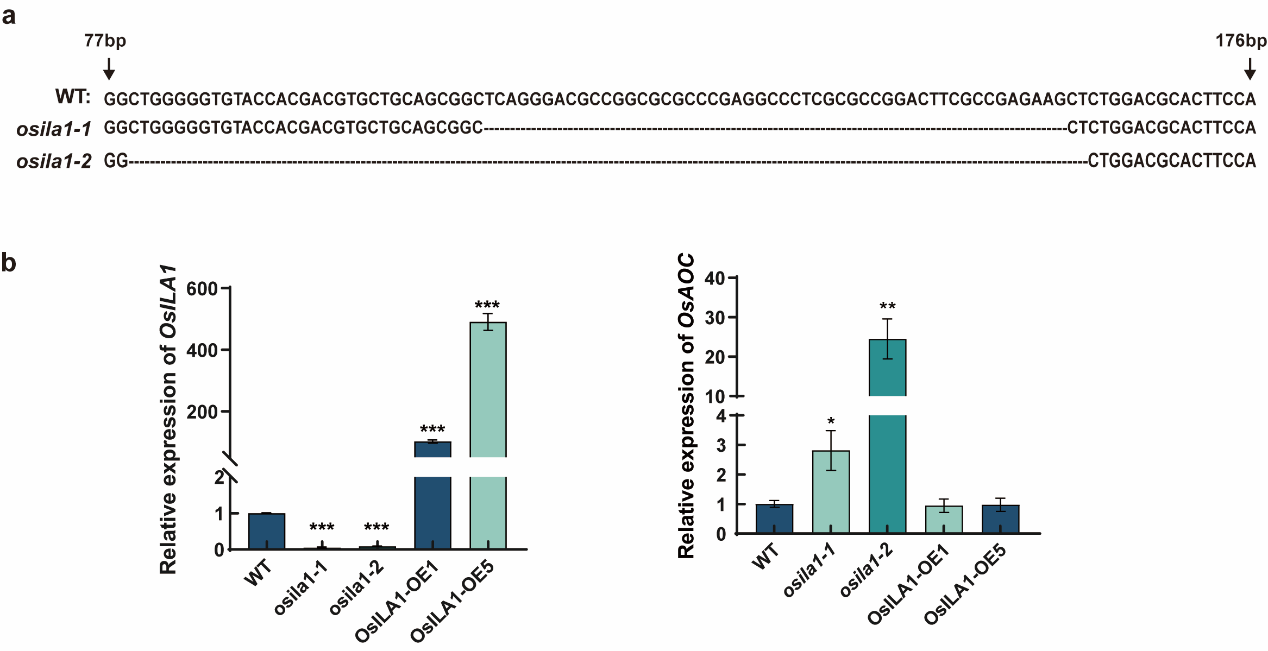
**

**Fig. S13 Construction of *OsILA1* transgenic lines.**

**a** Schematic diagram of the knockout of the *OsILA1* gene locus in *osila1-1* and *osila1-2* mutants, missing 110-159bp and 79-161bp within the coding sequences (CDSs) respectively. **b** Results of the qRT-PCR analysis of *OsILA1* and *OsAOC* transcription in OsILA1 transgenic lines. Error bars in **(b)** indicate ± SD (n = 3). The *p* values were determined by two-tailed unpaired Student’s *t*-test, ^∗^*p* < 0.05, ^∗∗^*p* < 0.01, ^∗∗∗^*p* < 0.001.

**
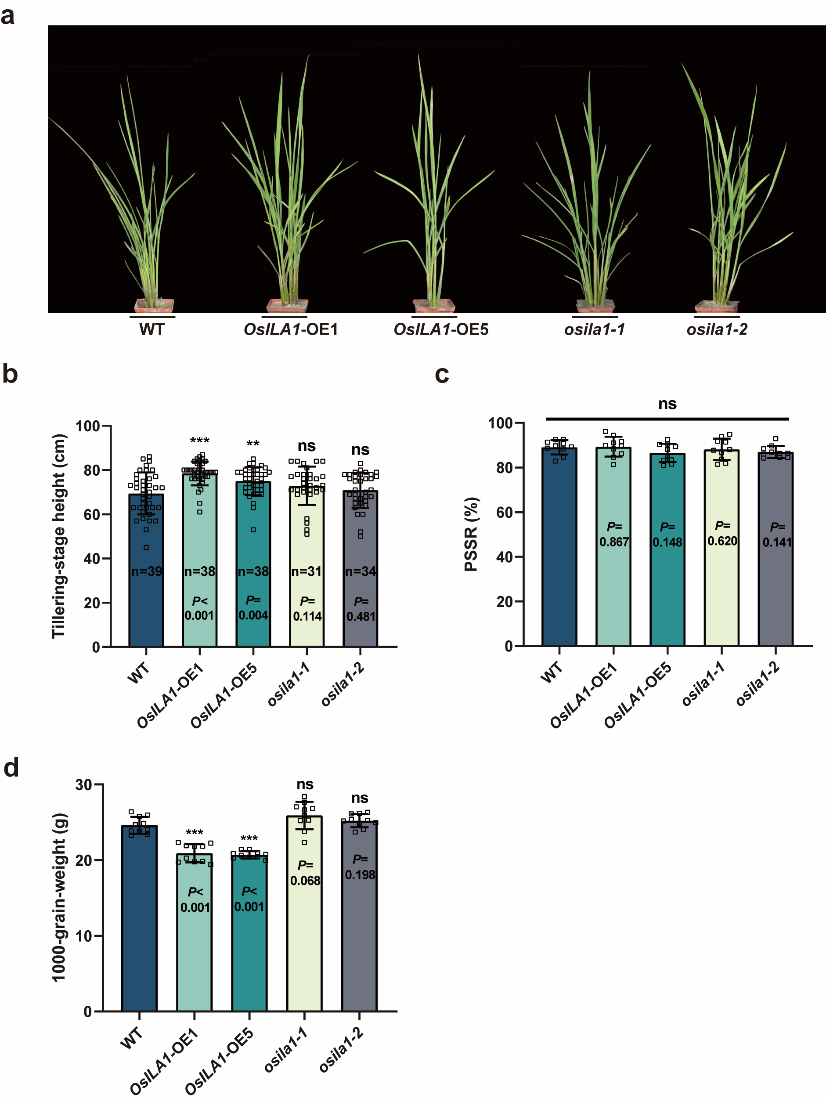
**

**Fig. S14 Agronomic traits of OsILA1 transgenic lines.**

**(a)** The tillering-stage of OsILA1 transgenic lines in the field. **(b-d)** Statistical results of tillering-stage height, panicle seed setting rate (n =10), and 1000-grain weight of field-grown plants (n =10). Error bars in **(b-d)** indicate ± SD. The p values were determined by two-tailed unpaired Student’s t-test, *^∗∗^p* < 0.01, *^∗∗∗^p* < 0.001.


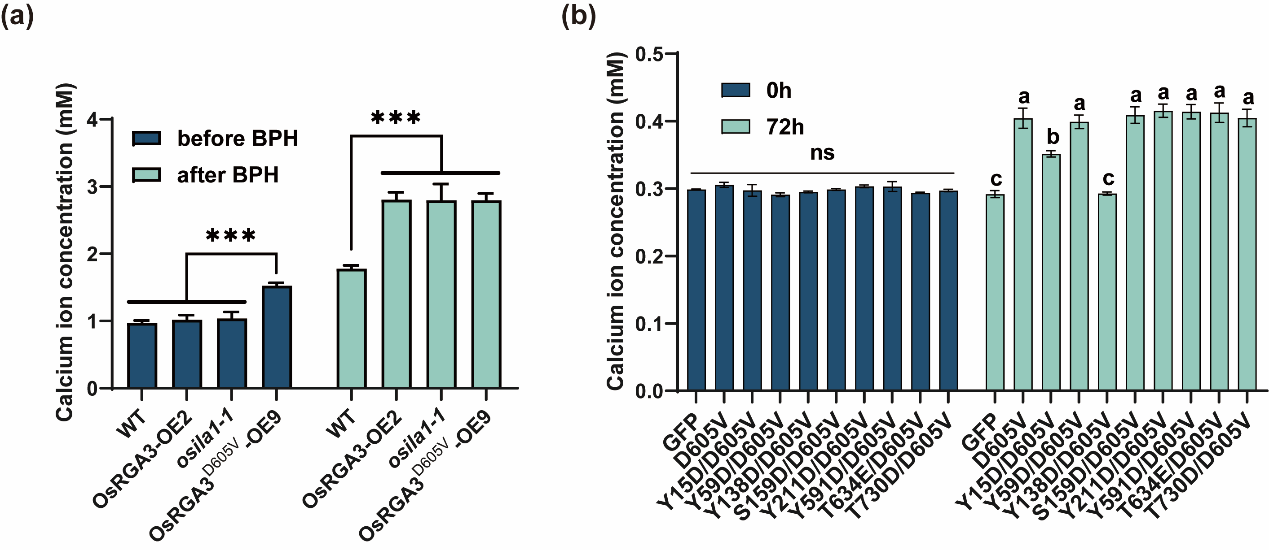


**Fig.** **S15 Ca^2+^ concentration detection in *Nb* leaves and rice stems**

**a** The calcium ion concentration in rice stems before and after BPH infestation. b The calcium ion concentration in *Nb* leaves 72 hours after the different protein fragments expression. Error bars indicate ± SD (n = 3). The *p* values in **(a)** were determined by two-tailed unpaired Student’s *t*-test, ^∗∗∗^*p* < 0.001. The *p* values in **(b)** were determined by one-way ANOVA with two-sided Tukey’s HSD test and indicated with different letters, *p*<0.01.

**Table S1** All primer sequences used in this study

| actin-F | AGGAATGGAAGCTGCGGGTAT |
| --- | --- |
| actin-R | GCAGGAGGACGGCGATAACA |
| UBQ-F | AACCACTTCGACCGCCACT |
| UBQ-R | GTTCGATTTCCTCCTCCTTCC |
| OsRGA3-qpcr-F | GCACAGCTGCTTGCTTCATT |
| OsRGA3-qpcr-R | TCCATGGTCCTGAAACACCG |
| OsRGA3-sgRNA-F | TGCACTACTTGCTCTTTCAAA |
| OsRGA3-sgRNA-R | AAAAACAACAGCATAGTGTG |
| OsUG-F | TTCTGGTCCTTCCACTTTCAG |
| OsUG-R | ACGATTGATTTAACCAGTCCATGA |
| MoPot2-F | ACGACCCGTCTTTACTTATTTG |
| MoPot2-R | AAGTAGCGTTGGTTTTGTTGGAT |
| OsRGA3-F | ATGGCGGAAGCTGCACTACTTG |
| OsRGA3-R | GGACCACTCGTACACACGAAGC |
| OsRGA3D605V-F | AGCTTGGCAGGGTTAGTACATGCAGCATGCATGTC |
| OsRGA3D605V-R | AGAGCCAGCTCTCTCAGAATGACATGCATGCTGCA |
| OsRGA3-CC-F | GCGGCCATGGTTGCTAAATCC |
| OsRGA3-CC-R | AAGTGTTGTCTGTTATTCTCCAT |
| OsRGA3-NB-F | CTAATGAAAGATGAGGATCTTGTGG |
| OsRGA3-NB-R | CTTTTCCACAAAACCTTCCGCAATCC |
| OsRGA3-LRR-F | AAAGGGGACAGCACTCTGGAGGAG |
| OsILA1-F | ATGGACCACGGCGGTCAGGTCTCTCC |
| OsILA1-R | ATGGACCTTCCCAAAAGTAAAGAAGC |
| OsILA1-qpcr-F | AGATATTGATGCGAGTGA |
| OsILA1-qpcr-R | TAAGGGCTGTGGTAGAG |
| OsILA1-sgRNA-F | TCGAACAGGTCGGCGATGGA |
| OsILA1-sgRNA-R | GGCCCTGATTCGGATTCACAGG |
| OsRGA3-P1-F | GAAGCTGCACTACTTGCTCTTTCAAAGATTGGTTCCGATG |
| OsRGA3-P2-R | TGCGATCCATCCCTTCAGGAGCTCATCGCTGAGGTCAGAG |
| OsRGA3-P2-F | CAGCGATGAGCTCCTGAAGGGATGGATCGCAGAGGTGCGA |
| OsRGA3-P3-R | GGTGACTTGAGTAGGAATAAGCTGACTGACAGGGTCAACC |
| OsRGA3-P3-F | CTGTCAGTCAGCTTATTCCTACTCAAGTCACCATCTCTGA |
| OsRGA3-P4-R | CACAAGATCCTCATCTTTCATTAGTCGGGGAAAATCGTAC |
| OsRGA3-P4-F | TCCCCGACTAATGAAAGATGAGGATCTTGTGGGGATGGAG |
| OsRGA3-P5-R | CACATTAATGTCAAATTTCTCCTTATGTTGCTCATCTACA |
| OsRGA3-P5-F | ATAAGGAGAAATTTGACATTAATGTGTGGCTTACTGTGTC |
| OsRGA3-P6-R | TGCTGCATGTACTAACCCTGCCAAGCTCATCATTATCCAC |
| OsRGA3-P6-F | ATGATGAGCTTGGCAGGGTTAGTACATGCAGCATGCATGA |
| OsRGA3-P7-R | GTGTCCATCGACATGCTGACAGGCGACGCACATTTTCGTT |
| OsRGA3-P7-F | TGTGCGTCGCCTGTCAGCATGTCGATGGACACACACAGAA |
| OsRGA3-P8-R | TAATTTTTCTATCTTGGTTGACTTAGCATCAAGATCTTGA |
| OsRGA3-P8-F | GATGCTAAGTCAACCAAGATAGAAAAATTACCTAGTGGGA |
| OsPR5-qpcr-F | CAACAGCAACTACCAAGTCGTC |
| OsPR5-qpcr-R | CAAGGTGTCGTTTTATTCATCAAC |
| OsPR10-qpcr-F | TCCTGTGTGGCCAAGCTCAA |
| OsPR10-qpcr-R | CAGGGTGAGCGACGAGGTAG |
| OsJAZ8-qpcr-F | GTTACCCACCTCAGCCTCAC |
| OsJAZ8-qpcr-F | TTTATACGGCGAAACCGAAC |
| OsAOC-qpcr-F | TCGTCCCCTTCACCAACAAG |
| OsAOC-qpcr-R | CTGTAGATGGCCTCGTAGCG |
